# Reduced polyphenol oxidase gene expression and enzymatic browning in potato (*Solanum tuberosum* L.) with artificial microRNAs

**DOI:** 10.1186/1471-2229-14-62

**Published:** 2014-03-11

**Authors:** Ming Chi, Basdeo Bhagwat, W David Lane, Guiliang Tang, Yinquan Su, Runcang Sun, B Dave Oomah, Paul A Wiersma, Yu Xiang

**Affiliations:** 1 College of Forestry, Northwest A & F University, Yangling, Shaanxi, China; 2 Agriculture and Agri-Food Canada, Pacific Agri-Food Research Centre, Summerland, British Columbia V0H 1Z0, Canada; 3 Department of Biological Sciences, Michigan Technological University, Houghton, MI 49931, USA

**Keywords:** Artificial microRNA (amiRNA), Enzymatic browning, Polyphenol oxidase gene family, *Solanum tuberosum* L

## Abstract

**Background:**

Polyphenol oxidase (PPO), often encoded by a multi-gene family, causes oxidative browning, a significant problem in many food products. Low-browning potatoes were produced previously through suppression of PPO gene expression, but the contribution of individual PPO gene isoform to the oxidative browning process was unknown. Here we investigated the contributions of different PPO genes to total PPO protein activity, and the correlations between PPO protein level, PPO activity and tuber tissue browning potential by suppression of all previously characterized potato PPO genes, both individually and in combination using artificial microRNAs (amiRNAs) technology.

**Results:**

Survey of the potato genome database revealed 9 PPO-like gene models, named *StuPPO1 to StuPPO9* in this report. *StuPPO1*, *StuPPO2*, *StuPPO3* and *StuPPO4* are allelic to the characterized *POTP1/P2*, *POT32*, *POT33* and *POT72*, respectively. Fewer ESTs were found to support the transcriptions of *StuPPO5* to *StuPPO8. StuPPO9* related ESTs were expressed at significant higher levels in pathogen-infected potato tissues. A series of browning phenotypes were obtained by suppressing *StuPPO1 to StuPPO4* genes alone and in combination. Down-regulation of one or several of the PPO genes did not usually cause up-regulation of the other PPO genes in the transgenic potato tubers, but resulted in reduced PPO protein levels. The different PPO genes did not contribute equally to the total PPO protein content in the tuber tissues, with StuPPO2 accounting for ~ 55% as the major contributor, followed by StuPPO1, ~ 25-30% and StuPPO3 and StuPPO4 together with less than 15%. Strongly positive correlations between PPO protein level, PPO activity and browning potential were demonstrated in our analysis. Low PPO activity and low-browning potatoes were produced by simultaneous down-regulation of *StuPPO2* to *StuPPO4*, but the greatest reduction occurred when *StuPPO1* to *StuPPO4* were all suppressed.

**Conclusion:**

*StuPPO1* to *StuPPO4* genes contributed to browning reactions in tuber tissues but their effect was not equal. Different PPO genes may be regulated independently reflecting their diversified functions. Our results show that amiRNAs can be used to suppress closely related members of highly conserved multi-gene family. This approach also suggests a new strategy for breeding low-browning crops using small DNA inserts.

## Background

Polyphenol oxidase (PPO) is nearly ubiquitous in angiosperms and belongs to a class of copper-binding enzymes that catalyze the oxidation of phenolics to quinones. The subsequent non-enzymatic polymerization of the quinones leads to formation of brown pigments that are the cause of post-harvest deterioration and loss of quality in many economically important crops [1,2]. Losses caused by the browning resulting from PPO catalyzed-oxidations probably account for 50% of the losses of industrial production of fruits and vegetables [3]. Several reports have described reduced browning reaction in crops by suppression of PPO gene expression using transgenic transformation with PPO gene fragments in configurations such as sense, antisense or double-stranded RNA [4-10]. Those approaches functioned by establishing an RNA silencing mechanism guided by a population of heterogeneous small interfering RNAs (siRNAs) [11]. Inevitably, the whole PPO gene family in the transgenic hosts was targeted because PPO genes are members of a multi-gene family with highly conserved gene sequences [2]. Because of this, it has been difficult to assess the contribution made by the individual PPO gene(s) to the oxidative browning process in different tissues. In addition to the undesired browning activity, PPOs appear to play important roles in signal transduction, stress and defense response throughout plant growth and development, but the specific PPO gene members involved in the different functions has not been elucidated. In potato (*Solanum tuberosum* L.), five PPO genes, namely *POTP1* (GenBank ID: M95196), *POTP2* (M95197), *POT32* (U22921), *POT33* (U22922) and *POT72* (U22923), were previously identified and characterized [1,12]. The nucleotide sequences of *POTP1* and *POTP2* are over 97% identical. *POTP1/P2*, *POT32*, *POT33* and *POT72* share 70-82% nucleotide homology. A previous study based on RNA Northern blot analysis revealed that *POTP1* and *POTP2* genes were expressed mainly in potato leaves and flowers, *POT32* and *POT33* mRNA were detected mainly in tubers with the *POT32* being the major form throughout tuber development, and *POT72* gene was mainly expressed in the roots [1].

Artificial microRNA (amiRNA) technology is a newly developed approach for inducing loss of gene function in plants [13-16]. It utilizes microRNA (miRNA) gene backbones to express artificial small RNAs that are usually 21 nucleotides (nt) in length. The resultant amiRNAs join in RNA silencing pathways and guide silencing of the gene of interest [17]. One of the advantages of amiRNA strategy is that it generates a single type of small RNA population all with the same selective nucleic acid sequence. It provides a feasible method for either silencing an individual gene or simultaneously silencing or partially silencing a multi-gene family while at the same time minimizing the risk of unpredicted off-target effects [18]. The amiRNA strategy has been applied in functional genetics studies using model plants, such as *Arabidopsis* and also agricultural crops, such as rice, alfalfa and tomato in recent years [19-21]. However, there were fewer reports of the targeting of closely related members of multi-gene families [20,22].

Here we reported suppression of the characterized members of the PPO gene family, i.e. *POTP1/P2*, *POT32*, *POT33* and *POT72*, individually or in combination in potato using amiRNAs. This allowed us to investigate the contributions of the different PPO genes to the total PPO protein activity in potato tubers and to further understand the correlations between PPO protein level, PPO activity and tuber tissue browning potential. Our results show that amiRNAs can be applied to suppress the expression of individual members of a highly conserved gene family. A series of browning phenotypes resulted from the suppression of different PPO gene isoforms in potato. Our results also suggest a new strategy for developing low-browning or non-browning crops.

The PPO gene suppression research in this report started before the availability of the potato genome sequence data, but we recently surveyed the PPO gene family in the potato genome and discovered 9 PPO-like gene models. The PPO gene models are systematically named as *StuPPO1* to *StuPPO*9 (*Solanumtuberosum* polyphenol oxidase 1 to 9). *POTP1/P2*, *POT32*, *POT33* and *POT72* are considered allelic to *StuPPO1*, *StuPPO2*, *StuPPO3* and *StuPPO4*, respectively. For continuity in the systematic nomenclature in this report, we renamed *POTP1/P2*, *POT32*, *POT33* and *POT72* to *StuPPO1*, *StuPPO2*, *StuPPO3* and *StuPPO4*.

## Results

### Genome-wide survey of PPO-like gene models in *Solanum tuberosum*

A genome-wide search of the recent *S. tuberosum* whole genome database in the US Joint Genome Institute (http://www.jgi.doe.gov) reveals 9 PPO-like gene models, tentatively named *StuPPO1 to StuPPO9* in this report (Table 1, Additional file 1). The *StuPPO1* to *StuPPO8* genes are aligned on chromosome 8, and *StuPPO9* is located on chromosome 2. *StuPPO1* and *StuPPO6* are in close proximity to each other in a 47-kb region, while *StuPPO2*, *StuPPO3*, *StuPPO4*, *StuPPO5*, *StuPPO7* and *StuPPO8* are clustered in tandem in a 144-kb region. The two regions are separated by a distance of 2,072-kb on chromosome 8. Analysis of the deduced amino acid sequences of the major peptides encoded by the PPO-like genes suggests that the predicted proteins all contain three typical PPO protein domains: the tyrosinase domain (pfam00264), the PPO1_DWL domain (pfam12142) and the PPO1_KFDV domain (pfam12143) [23,24], but the tyrosinase domains with the putative StuPPO5 and StuPPO7 peptides are shorter than the others and incomplete (Additional file 1: Part B). *StuPPO1*, *StuPPO2*, *StuPPO3* and *StuPPO4* are possibly allelic to the characterized *POTP1/P2*, *POT32*, *POT33* and *POT72*, respectively, considering that the nucleotide sequences between the potential alleles are 95-99% identical (Table 1, Additional file 1). Numerous ESTs were found from different developmental potato tissues for *StuPPO1* to *StuPPO4* (Additional file 1: Part A and Part C). Surprisingly, *StuPPO1* appears to be the only possible allele to the *POTP1* and *POTP2* genes, and no duplication of the *StuPPO1* locus was observed by searching the *S. tuberosum* genome sequence. *StuPPO5*, *StuPPO6* and *StuPPO7* are three novel PPO-like gene models predicted from this *S. tuberosum* genome sequence analysis. However, only few EST fragments (0 to 3) that probably relate to the potential transcripts of these three genes were found, and the ESTs cover only fragmental regions of the putative transcripts (Additional file 1: Part A and Part C). The low prevalence in EST databases suggests that *StuPPO5*, *StuPPO6* and *StuPPO7* may be expressed at very low levels in *S. tuberosum. StuPPO8* and *StuPPO9* are the only PPO-like gene models with introns. No EST from *S. tuberosum* EST databases was found for *StuPPO8*, suggesting that this gene sequence is normally not transcribed. *StuPPO9* is the only PPO-like gene model that is not clustered with the others on chromosome 8, but is independently located on chromosome 2. A number of ESTs were found for *StuPPO9*, but all of the ESTs were revealed in the cDNA libraries from the tissues of *in vitro* cultured potato callus (DBLINK ID: LIBEST_015047), abiotic stress treated leaf and root (LIBEST_015048) [25], or pathogen-infected leaf and tuber (LIBEST_008810, LIBEST_009854, LIBEST_015324, LIBEST_015920, LIBEST_017649, and LIBEST_025550) (Additional file 1: Part A and Part C) [26-28]. The expression data of the supporting ESTs of *StuPPO9* were mostly not available because the gene model was not annotated previously and most reports focused on annotated genes. However, at least three of the *StuPPO9* related ESTs (GenBank ID: CK640809, CO267905 and GT888802) were found to express differentially in the pathogen-infected potato tissues (Additional file 1: Part C). For example, the expression level of the CK640809 was induced 3- to 14-fold higher in potato (cultivar Indira and Bettina) leaf tissue that was inoculated with fungus *Phytophthora infestans* (See the Table four in reference [27]. The CO267905 showed over 2-fold induction in the potato (cv. R-gene-free potato clone 386209.10) leaf tissue infected with *P. infestans* at 24 hours post-inoculation (hpi) and its expression level was over 17-fold higher at 48 hpi (See the Table one in reference [28]). The relative expression level of GT888802 was about 3-fold higher in potato (cv. Spunta) tubers inoculated with fungus *Fusarium eumartii* at 24 hpi (Table S2 in reference [26]). These data imply that *StuPPO9* is probably an inducible PPO gene expressed in response to disease defense and cell rescue [28].

**Table 1:** List of predicted potato PPO gene models

**Tentative gene name (in this report)**	**Locus location**	**Number of intron**	**Predicted transcript name assigned by potato genome sequencing consortium (PSGC)**	**Possible Allele (GenBank ID, nucleotide sequence identity%)**
*StuPPO1*	chr08, 30458794..30460741	0	PSGC003DMT400076054	*POTP1* (M95196, 97.3%)/*POTP2* (M95197, 97.9%)/XM_006355177 (100%)
*StuPPO2*	chr08, 32672194..32674192	0	PSGC0003DMT400048684	*POT32* (U22921, 96.7%)/XM_006365321 (95.5%)
*StuPPO3*	chr08, 32687330..32689280	0	PSGC0003DMT400048681	*POT33* (U22922, 94.7%)/XM_006365320 (100%)
*StuPPO4*	chr08, 32667904..32669792	0	PSGC0003DMT400048685	*POT72* (U22923, 96.8%)
*StuPPO5*	chr08, 32591830..32593339	0	PSGC0003DMT400048692	XR_183056 (84.6%)
*StuPPO6*	chr08, 30504049..30505788	0	PSGC0003DMT400076055	XM_004245989 (89.1%)
*StuPPO7*	chr08, 32577708..32579291	0	PSGC0003DMT400048703	XR_183056 (84.8%)
*StuPPO8*	chr08, 32703818..32721729	2	PGSC0003DMT400048679	XM_006365329 (100%)
*StuPPO9*	chr02, 55593718..55596019	1	not available	XM_006347021 (100%)

### Generation and selection of amiRNA-expressed transgenic potatoes

**Figure 1:**
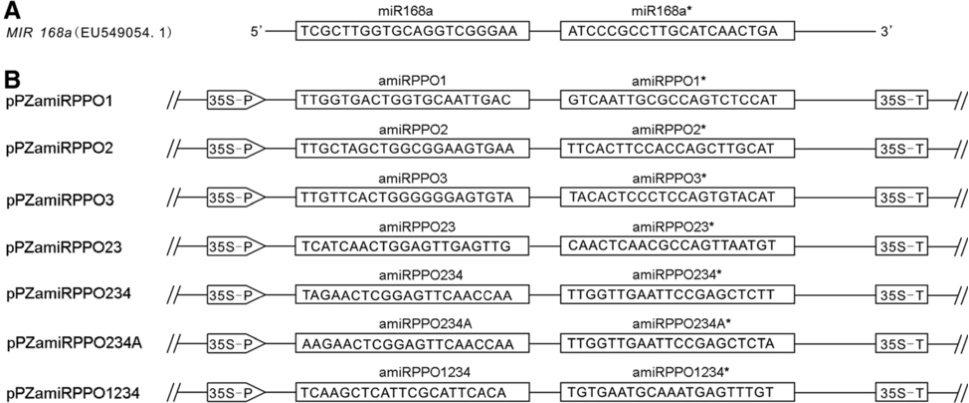
**Diagrammatic representation of artificial microRNA constructs. (A)** Linear structure of the miR168a primary transcript gene (*MIR168a*, nt 120 to 355, GenBank Accession No. EU549054.1). Sequences of the miR168a and its complementary region (illustrated as approximately miR168a*) in the gene are displayed in the boxes. **(B)** Structure of the binary vectors for expression of amiRNAs. Construct names are indicated at the left. The sequences of the designed amiRNA and its complementary region (approximately amiRNA*) are displayed in the boxes. 35S-P, CaMV 35S promoter. 35S-T, CaMV 35S terminator.

Seven different amiRNAs were designed to target the characterized PPO genes, namely *StuPPO1* (previously named *POTP1/P2*), *StuPPO2* (previously, *POT32*), *StuPPO3* (previously, *POT33*) and *StuPPO4* (previously, *POT72*) in *S. tuberosum*[1,12], with amiRNA sequences complementary to either a specific gene or multiple targets by choosing the appropriate 21-bp region of the corresponding PPO genes (Table 2). The amiRNA sequences were incorporated in an *Arabidopsis thaliana* miR168a gene backbone built in a plant binary vector (Figure 1), [29,30]. From transformation of thousands of explants, 8 to 10 transgenic potato lines for each amiRNA construct were selected and propagated for molecular genetic screening and analysis (Table 3). Significant down-regulation of the targeted gene expression was detected in a number of the resulting transgenic potato lines (Table 3). Based on the initial real-time quantitative reverse transcription PCR (qRT-PCR) assay of the *in vitro* cultured potato plants, the transcript levels of the *StuPPO1* genes were reduced by 68 to 98% in six amiRPPO1 series transgenic lines (clones). The amiRPPO1 series expressed artificial miRNA - amiRPPO1 designed to target the *StuPPO1* gene. Similarly, one amiRPPO2 series line (target: *StuPPO2*), two amiRPPO3 series lines (target: *StuPPO3*), five amiRPPO23 series lines (targets: *StuPPO2* and *StuPPO3*), two amiRPPO234 series lines (targets: *StuPPO2*, *StuPPO3* and *StuPPO4*), three amiRPPO234A series lines (targets: *StuPPO2*, *StuPPO3* and *StuPPO4*) and five amiRPPO1234 series lines (targets: *StuPPO1*, *StuPPO2*, *StuPPO3* and *StuPPO4*) showed substantial reduction of the target gene transcript level(s) (Table 3). Mature amiRNAs were detected by reverse transcription PCR (RT-PCR) in the selected amiRPPO1, amiRPPO3, amiRPPO23, amiRPPO234A and amiRPPO1234 series lines (Table 3, Additional file 2: Figure S1). However, no mature amiRNAs were revealed by RT-PCR in the 10 lines of the amiRPPO2 series nor the 10 clones of the amiRPPO234 series (Table 3 and data not shown). The amiRPPO2- and amiRPPO234- transgenic lines were therefore excluded from further evaluations. In addition, small RNA Northern blots were previously done to detect amiRNA expression of the multiple transgenic lines listed in Table 3, including amiRPPO1-7 and −12 (previously named as amiR-POTP1/P2, L7 and L12), amiRPPO2-12, −19 (previously, amiR-POT32, L12 and L19), amiRPPO3-8 and −15 (previously, amiR-POT33, L8 and L15) and amiRPPO234-9 and −10 (previously, amiR-POT32/33/72, L9 and L10) [30].

**Table 2:** List of the amiRNA constructs, amiRNA names, amiRNA target sequences and target genes

**Construct**	**amiRNA name**	**amiRNA sequence (5′ → 3′)**	Target sequence* (5′ → 3′)	**Gene name (GenBank accession ID)**
pPZamiRPPO1	amiRPPO1	UUGGUGACUGGUGCAAUUGAC	GUCAAUUGCACCAGUCACCAA	*StuPPO1/POTP1*/*P2* (XM_006355177/M95196/M95197)
pPZamiRPPO2	amiRPPPO2	UUGCUAGCUGGCGGAAGUGAA	UUCACUUCCGCCAGCUAGCAA	*StuPPO2/POT32* (U22921)
pPZamiRPPO3	amiRPPO3	UUGUUCACUGGGGGGAGUGUA	UACACUCCCCCCAGUGAACAA	*POT33* (U22922)
			U**U**CACUCCCCCCAGUGAAC**G**A	*StuPPO3* (XM_006365320)
pPZamiRPPO23	amiRPPO23	UCAUCAACUGGAGUUGAGUUG	CAACUCAACUCCAGUUGAUGA	*StuPPO2*/*POT32*
			CAACUCAACUCCAGUUGAUGA	*StuPPO3/POT33*
pPZamiRPPO234	amiRPPO234	UAGAACUCGGAGUUCAACCAA	UUGGUUGAACUCCGAGUUCUA	
			UUGGUUGAACUCCGAGUUCU**U**	*StuPPO2/POT32*
			UUGGUUGAACUCCGAGUUCU**U**	*StuPPO3* (XM_006365320)
			UUGGUUGAACUC**U**GAGUUCU**U**	*POT33* (U22922)
			UUGGUUGAACUCCGAGUUCU**U**	*StuPPO4*/*POT72* (U22923)
pPZamiRPPO234A	amiRPPO234A	AAGAACUCGGAGUUCAACCAA	UUGGUUGAACUCCGAGUUCUU	*StuPPO2*/*POT32*
			UUGGUUGAACUC**U**GAGUUCUU	*StuPPO3/POT33*
			UUGGUUGAACUCCGAGUUCUU	*StuPPO4*/*POT72*
pPZamiRPPO1234	amiRPPO1234	UCAAGCUCAUUCGCAUUCACA	UGUGAAUGCGAAUGAGCUUGA	
			UGUGAAUGCG**G**AUGAGCUUGA	*StuPPO1/POTP1*/*P2*
			UGUGAAUGC**A**AAUGAGCUUGA	*StuPPO2* (XM_006365321)
			UGUGAAUGCGAAUGAGCUUGA	*POT32* (U22921)
			UGUGAAUGCGAAUGAGCUUGA	*StuPPO3/POT33*
			UGUGAAUGCGAAUGAGCUUGA	*StuPPO4/POT72*

Note: * The non-identical nucleotides, compared to the target sequence, are marked in **bold**. The homologous sequences of the predicated PPO-like gene models (*StuPPO1* to *StuPPO9*) to all amiRNA target sequences are aligned in the Additional file 1: Part D.

**Table 3:** Screening amiRNA-expressed transgenic potato lines

**Transgenic line**	**Kan**	**Insert**	**Relative expression level of target gene**				**amiRNA**
			**POTP1/P2**	**POT32**	**POT33**	**POT72**	
amiRPPO1-2	+	+	0.02 ± 0				+
amiRPPO1-3	+	+	0.06 ± 0				+
amiRPPO1-7	+	+	0.14 ± 0				+
amiRPPO1-8	+	+	0.03 ± 0				n.t.
amiRPPO1-9	+	+	0.15 ± 0.01				n.t.
amiRPPO1-10	+	n.t.	n.t.				n.t.
amiRPPO1-12	+	+	0.34 ± 0				+
amiRPPO1-13	+	n.t.	n.t.				n.t.
WT	-	-	0.99 ± 0.08				-
amiRPPO2-1	+	n.t.		n.t.			-
amiRPPO2-4	+	n.t.		1.42 ± 0.12			-
amiRPPO2-9	+	+		n.t.			-
amiRPPO2-12	+	+		1.03 ± 0.02			-
amiRPPO2-13	+	n.t.		n.t.			-
amiRPPO2-15	+	n.t.		0.83 ± 0.11			-
amiRPPO2-16	+	+		0.45 ± 0.03			-
amiRPPO2-17	+	n.t.		n.t.			-
amiRPPO2-18	+	+		1.07 ± 0.04			-
amiRPPO2-19	+	+		0.99 ± 0.03			-
WT	-	-		1.00 ± 0.01			-
amiRPPO3-8	+	+			1.29 ± 0.23		+
amiRPPO3-9	+	n.t.			n.t.		n.t.
amiRPPO3-12	+	+			0.66 ± 0.12		+
amiRPPO3-13	+	n.t.			0.97 ± 0.17		n.t.
amiRPPO3-14	+	+			n.t.		n.t.
amiRPPO3-15	+	+			n.v.		+
amiRPPO3-16	+	n.t.			1.31 ± 0.40		n.t.
amiRPPO3-17	+	+			n.t.		n.t.
amiRPPO3-18	+	+			n.t.		n.t.
WT	-	-			1.00 ± 0		-
amiRPPO23-3	+	+		n.t.	n.t.		n.t.
amiRPPO23-4	+	n.t.		1.03 ± 0.11	1.28 ± 0.29		n.t.
amiRPPO23-5	+	+		0.32 ± 0.01	0.44 ± 0.08		+
amiRPPO23-7	+	+		0.28 ± 0.03	0.19 ± 0.01		+
amiRPPO23-8	+	+		0.42 ± 0	0.01 ± 0		+
amiRPPO23-9	+	+		0.07 ± 0.01	0.01 ± 0		+
amiRPPO23-14	+	n.t.		n.t.	n.t.		n.t.
amiRPPO23-15	+	n.t.		0.23 ± 0.07	0.00 ± 0		n.t.
amiRPPO23-16	+	n.t.		n.t.	n.t.		n.t.
WT	-	-		1.00 ± 0.08	1.00 ± 0.12		-
amiRPPO234-3	+	n.t.		n.t.	n.t.	n.t.	-
amiRPPO234-4	+	n.t.		n.t.	n.t.	n.t.	-
amiRPPO234-5	+	+		0.87 ± 0.06	0.92 ± 0.14	1.03 ± 0.17	-
amiRPPO234-6	+	n.t.		0.98 ± 0.12	3.39 ± 0.47	1.50 ± 0.08	-
amiRPPO234-7	+	+		1.22 ± 0.16	1.16 ± 0.09	1.04 ± 0.17	-
amiRPPO234-8	+	+		1.66 ± 0.02	0.71 ± 0.04	0.69 ± 0.02	-
amiRPPO234-9	+	+		0.47 ± 0.04	0.40 ± 0.09	0.50 ± 0.13	-
amiRPPO234-10	+	+		0.61 ± 0.03	0.61 ± 0.09	0.54 ± 0.21	-
amiRPPO234-11	+	n.t.		1.35 ± 0.16	0.83 ± 0.17	1.16 ± 0.13	-
amiRPPO234-13	+	n.t.		n.t.	n.t.	n.t.	-
WT	-	-		1.00 ± 0.01	1.00 ± 0.08	1.00 ± 0.01	-
amiRPPO234A-2	+	n.t.		1.09 ± 0.20	1.53 ± 0.19	1.26 ± 0.13	n.t.
amiRPPO234A-4	+	+		0.34 ± 0.05	0.43 ± 0.07	0.04 ± 0.01	+
amiRPPO234A-6	+	+		0.09 ± 0.01	0.02 ± 0	0.05 ± 0.01	+
amiRPPO234A-10	+	+		1.07 ± 0.03	0.77 ± 0.25	0.47 ± 0.02	+
amiRPPO234A-13	+	+		n.t.	n.t.	n.t.	n.t.
amiRPPO234A-14	+	+		0.58 ± 0	0.22 ± 0.01	0.15 ± 0.03	+
amiRPPO234A-15	+	n.t.		1.25 ± 0.18	0.8 ± 0.12	1.34 ± 0.16	n.t.
amiRPPO234A-16	+	n.t.		0.83 ± 0.12	1.21 ± 0.09	0.60 ± 0.07	n.t.
WT	-	-		0.99 ± 0.08	1.01 ± 0.18	0.99 ± 0.05	-
amiRPPO1234-1	+	n.t.	n.t.	n.t.	n.t.	n.t.	n.t.
amiRPPO1234-2	+	+	0.52 ± 0.07	0.30 ± 0.03	0.46 ± 0.01	0.34 ± 0.08	+
amiRPPO1234-3	+	+	0.43 ± 0.04	0.19 ± 0.06	0.07 ± 0	0.43 ± 0.1	n.t.
amiRPPO1234-6	+	+	0.27 ± 0.03	0.20 ± 0.04	0.19 ± 0	0.2 ± 0.04	+
amiRPPO1234-7	+	n.t.	n.t.	n.t.	n.t.	n.t.	n.t.
amiRPPO1234-8	+	n.t.	n.t.	n.t.	n.t.	n.t.	n.t.
amiRPPO1234-9	+	+	n.t.	n.t.	n.t.	n.t.	n.t.
amiRPPO1234-11	+	n.t.	0.15 ± 0.01	0.34 ± 0.11	1.85 ± 0.17	0.08 ± 0.01	n.t.
amiRPPO1234-12	+	+	0.28 ± 0.03	0.66 ± 0.02	0.47 ± 0.03	0.18 ± 0.01	+
amiRPPO1234-16	+	n.t.	0.42 ± 0.03	0.14 ± 0.03	0.15 ± 0	0.08 ± 0.06	n.t.
WT	-	-	1.00 ± 0.09	1.00 ± 0.1	1.00 ± 0.19	1.00 ± 0.07	-

Note: Kan, is selection of transgenic lines with Kanamycin (100 mg/L); +, Kanamycin resistant; −, Kanamycin susceptible (such as for WT). Insert, is transgene insertion into the chromosome(s) detected by PCR; +, positive in PCR detection; −, negative in PCR detection; n.t., not test. Relative expression level of target gene, was assayed using *in vitro* cultured potato leaf and stem tissues by qRT-PCR; the data is the average of three technical repeats and their standard deviation of using one original sample from each transgenic line; n.v., no value; n.t. not test. amiRNA, is detection of mature amiRNAs by RT-PCR; +, detectable (positive); −, not detectable (negative); n.t., not test.

**Figure 2:**
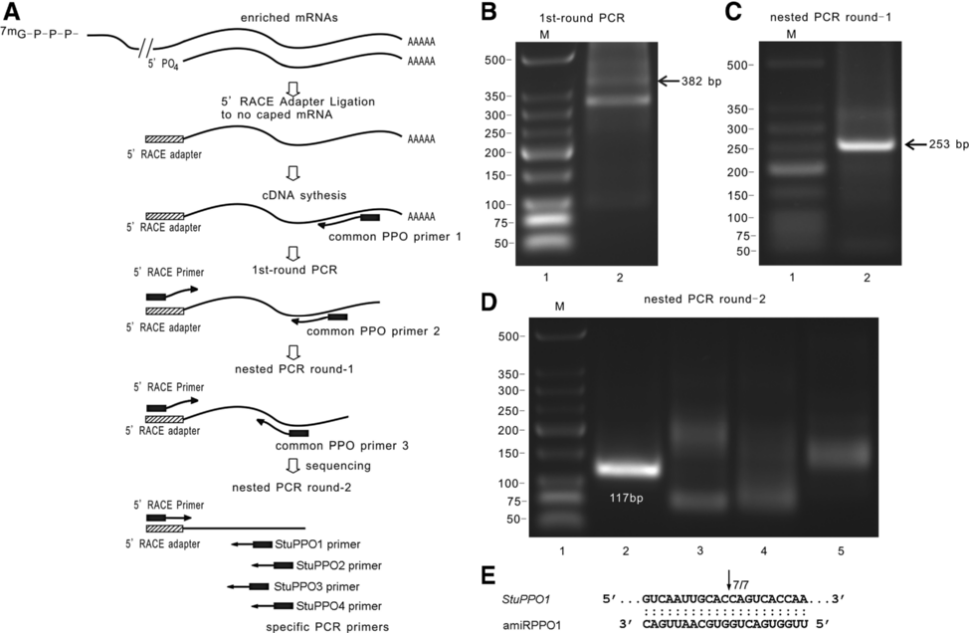
**Detection of cleaved PPO gene mRNAs by 5′ RACE-PCR. (A)** Schematic diagram of a strategy for detecting the truncated but not the 5′-capped PPO gene mRNAs in amiRNA-expressed transgenic plants. **(B)** RACE-PCR (1st-round PCR). Lane 1, DNA ladder (NEB, Cat# N0474S); lane 2, 1st-round PCR result from the enriched young leaf poly(A)^+^ RNA of transgenic line amiRPPO1-12. The line with an arrowed end indicates the band of the expected size. **(C)** Nested PCR round-1 (using the RACE-PCR product as the template). Lane 1, DNA ladder; lane 2, nested PCR-1 result; the line with an arrowed end indicates the band of the expected size. **(D)** Nested PCR round-2 (using nested PCR-1 product as template). Lane 1, DNA ladder; lane 2, PCR for detection of truncated *StuPPO1* gene mRNA; amplified band size as indicated. Lane 3, PCR for detection of truncated *StuPPO2* gene mRNA; the faint bands were non-specific amplification. Lane 4, PCR for detection of truncated *StuPPO3* gene mRNA; the faint bands were non-specific amplification. Lane 5, PCR for detection of truncated *StuPPO4* gene mRNA; the faint bands were non-specific amplification. **(E)** Determination of the target cleavage site of amiRPPO1 by sequencing the 253 bp of the nested-PCR-1 product (Figure 3C). The target sequences are aligned with the amiRPPO1 complementarily. The arrowed line indicates that 7 of the 7 clones (7/7) were the products cleaved at the expected site. See Additional file 3: Figure S2 and Additional file 4: Figure S3 for the full sequence analysis.

In plants, miRNA-guided RNA silencing has been shown to occur mostly through complementary cleavage of the targeted mRNA by Argonaute proteins [31]. Considering the highly complementary sequences between the designed amiRNAs and their target PPO genes, we used 5′-RACE PCR to detect the possible cleavage of the PPO gene transcript(s). Because of the multiple PPO gene members in potatoes, we developed a strategy for detecting all possible fragmented-mRNA of the PPO genes but not the 5′-capped mRNA (Figure 2A and see the Methods). As predicted, a 253-bp PCR product (including a 45-bp 5′-RACE adaptor) was revealed by the nested PCR round-1 from the enriched poly(A)^+^ RNA of the young leaves of line amiRPPO1-12 (Figure 2C). Sequence analysis demonstrated the fragment included two nearly identical sequences differing by one nucleotide (Additional file 3: Figure S2). Both were highly related to the *StuPPO1* gene (97 to 99% identity at nucleotide level) (Additional file 4: Figure S3). The first 10 nucleotides of the 5′-end of the sequences were complementary to the 5′-end of the designed amiRPPO1, indicating the products were from cleavage of the target *StuPPO1* mRNA and the cleavage site was between nucleotides 10 and 11 at the amiRPPO1 site (Figure 2E). The presence of the cleaved *StuPPO1* mRNA was also demonstrated by the specific nested PCR-2 (Figure 2D). The results suggested that the expressed amiRNAs in the transgenic plants functioned as the small RNAs that determined the silencing of the gene(s) of interest. The following transgenic lines were selected and propagated for further biological analysis, amiRPPO1-2, −3 and −12, amiRPPO3-12 and −15, amiRPPO23-5, −7 and −9, amiRPPO234A-4, −6 and −14, amiRPPO1234-2, −6 and −12 (Table 3).

### PPO gene expressions in transgenic potatoes

**Figure 3:**
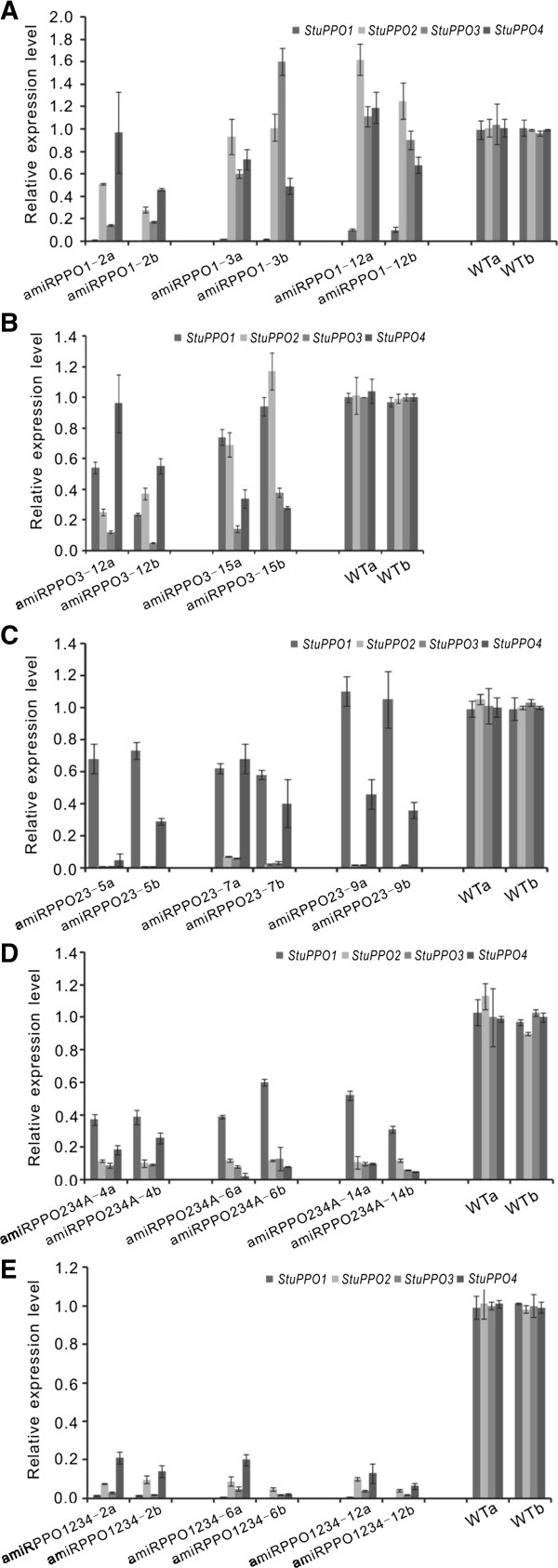
**Relative transcript levels of PPO genes in transgenic potato tuber tissues. (A)** Transgenic lines of series amiRPPO1. **(B)** Transgenic lines of series amiRPPO3. **(C)** Transgenic lines of series amiRPPO23. **(D)** Transgenic lines of series amiRPPO234A. **(E)** Transgenic lines of series amiRPPO1234. Each column represents the mean value obtained from qRT-PCR performed in triplicate on each biological sample. The bars indicate standard deviation. Two biological replicates (indicated as a and b) from each transgenic line were selected for the assay. Cyclophilin and ef1α genes were used as normalization references and non-transgenic potatoes (WT) were set as the control.

No growth abnormities occurred in the amiRNA-expressed transgenic plants under greenhouse conditions. Nor were the range of the tuber sizes and weights significantly different in either the transgenic or the wild types (Data not shown). Relative transcript levels of *StuPPO1*, *StuPPO2*, *StuPPO3* and *StuPPO4* genes in tuber tissues of the transgenic lines were assayed by qRT-PCR with the results illustrated in Figure 3. For lines amiRPPO1-2, −3 and −12, the expression of the target *StuPPO1* gene was suppressed by 90 to 99%. The mRNA levels of the non-targeted *StuPPO2* and *StuPPO3* in lines amiRPPO1-3 and amiRPPO1-12 were similar to that of the non-transgenic, wild types ‘WT’, potato controls, but the expression levels of the two genes (*StuPPO2* and *StuPPO3*) in line amiRPPO1-2 were unexpectedly reduced by 50% and 80%, respectively (Figure 3A). For lines amiRPPO3-12 and −15, the transcript of the target *StuPPO3* gene was reduced by over 75%. In addition, the non-targeted *StuPPO1* and *StuPPO2* gene mRNA levels also decreased by 50-60% in line amiRPPO3-12, but the two gene transcripts (*StuPPO1* and *StuPPO2*) in line amiRPPO3-15 were close to the level observed in the WT. The non-targeted *StuPPO4* gene mRNA was reduced by ~60% in line amiRPPO3-15 but the same gene mRNA level in line amiRPPO3-12 was almost the same as the WT (Figure 3B). For lines amiRPPO23-5, −7 and −9, the two targeted genes, *StuPPO2* and *StuPPO3* were almost completely silenced (> 95%) and the non-targeted *StuPPO1* gene was expressed at a level similar to the WT, but the *StuPPO4* gene mRNA level was also generally 50-80% lower (Figure 3C). For lines amiRPPO234A-4, −6 and −14, the mRNA levels of all three targets, *StuPPO2*, *StuPPO3* and *StuPPO4* were 75-95% lower than the WT, but the expression of the non-targeted *StuPPO1* gene was also reduced by an average of 50% (Figure 3D). For lines amiRPPO1234-2, −6 and −12, the mRNA levels of the targeted *StuPPO1*, *StuPPO2*, *StuPPO3* and *StuPPO4* genes were all suppressed by 85-99% (Figure 3E).

### PPO protein level

**Figure 4:**
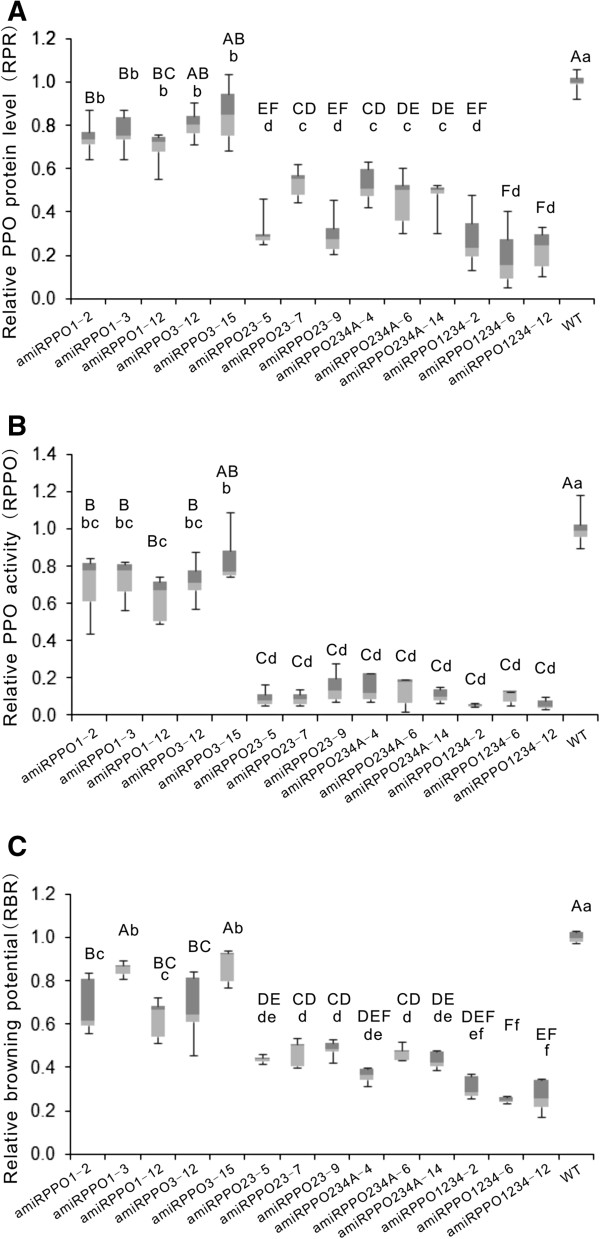
**Assay of PPO protein level, enzymatic activity and browning potential in transgenic potato tuber tissues. (A)** Relative PPO protein level (RPR). **(B)** Relative PPO activity (RPPO). **(C)** Relative browning potential (RBR). Each box plot presents the data from three biological and three technical replicates of the transgenic and non-transgenic (WT) potato tubers. All data are presented relative to the level of the WT. A line across the box indicates the median. The box indicates the 25th and 75th percentiles. Whiskers represent the maximum and minimum values. Different lower case letters indicate values are significantly different at P < 0.05 level; different capital letters indicate values are highly significantly different at P < 0.01 level based on Duncan’s Multiple Range Test.

Total PPO protein levels in the transgenic and non-transgenic potato tubers were analyzed using a semi-quantitative protein dot-blot assay. Figure 4A shows the values of the total PPO protein level in the transgenic potato tuber tissues relative to that in the non-transgenic wild types (relative PPO protein level, simply represented by ‘RPR’ in this report). The average RPR values for lines amiRPPO1-2, −3 and −12 ranged from 0.70 to 0.77, indicating total PPO protein levels in these transgenic potato tuber tissues were on average about 23-30% lower than those in the wild type. Lines amiRPPO3-12 and −15 showed a decline of 15-20% on average in their total PPO protein level, compared to the wild type. Average reductions of 45-70% in total PPO protein level were detected in lines amiRPPO23-5, −7 and −9, amiRPPO234A-4, −6 and −14, based on their average RPRs (0.30-0.55). The average RPRs for lines amiRPPO1234-2, −6 and −12 varied from 0.20 to 0.27, suggesting that total PPO protein concentrations in these transgenic tubers decreased on average by 73-80%, compared to the wild type (Figure 4A).

### PPO enzymatic activity

Figure 4B depicts the PPO enzymatic activities in the transgenic potato tuber tissues relative to those in the wild type (relative PPO activity, abbreviated to ‘RPPO’ in this report). Based on the RPPOs, the average PPO activity of lines amiRPPO1-2, −3 and −12 was 25-35% lower than that of the wild type. A reduction of 15-25% in PPO activity was observed in lines amiRPPO3-12 and −15. PPO activities in lines amiRPPO23-5, −7 and −9, amiRPPO234A-4, −6 and −14, amiRPPO1234-2, −6 and −12 were in a similar range, about 75-95% less than those of the non-transgenic controls (Figure 4B).

### Browning potential and browning phenotype

**Figure 5:**
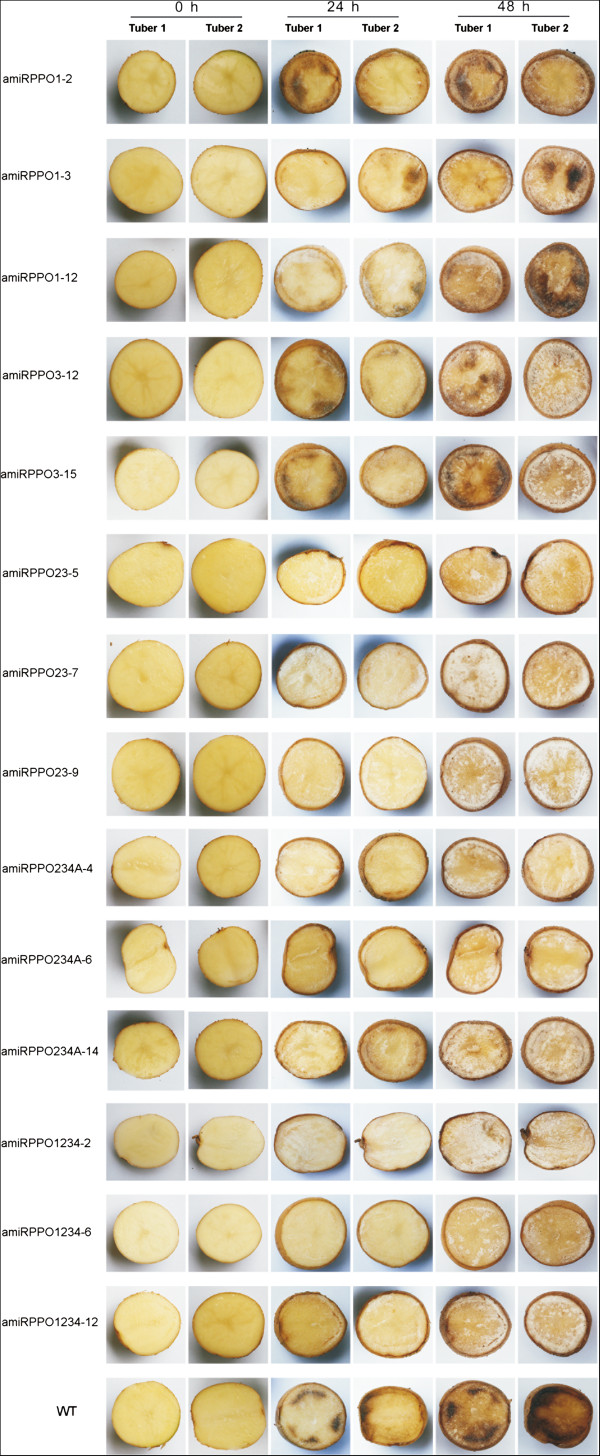
**Browning phenotypes.** Potato tubers from each transgenic line and wild type were randomly selected, cut into approximately two equal sections and exposed to air/oxygen at room temperature (~25°C). Images were taken at 0, 24 and 48 hour post air exposure. Browning or blackening typically started from vascular ring region and advanced to the medullary tissue with the air-exposure time.

Browning was used to measure the potential of phenolic oxidation after mechanical release of PPO proteins from their storage site in plastids [32]. Browning potential of the transgenic potato tuber was compared to that of their non-transgenic wild types (relative browning potential, abbreviated to ‘RBR’ in this report) and the results are shown in Figure 4C. The RBRs for lines amiRPPO1-2, −3 and −12, amiRPPO3-12 and −15 ranged between 0.65 and 0.90, suggesting that browning potentials of these transgenic lines were about 10-35% lower than those of the WT. Browning potentials of lines amiRPPO23-5, −7 and −9, amiRPPO234A-4, −6 and −14 were reduced by ~50-65% based on their RBR values of ~0.35-0.50. The ranges of 0.25-0.35 in RBR for lines amiRPPO1234-2, −6 and −12 indicated that the browning potential of these transgenic lines was about 65-75% lower than the comparable wild type (Figure 4C). The browning potential results were relatively consistent with the series of visible browning phenotypes displayed after air-exposure of the freshly sectioned potato tubers at room temperature (Figure 5). Browning or blackening tissues developed on the sectioned tuber surfaces, typically starting from the vascular ring region and advancing to the medulla with increased exposure time to oxygen in the air. The wild type tubers developed brown tissues more quickly, over a larger area and more severely than in the transgenic tubers. Among the different transgenic types, the amiRPPO1 and amiRPPO3 series lines showed relatively stronger browning phenotype, followed by the amiRPPO23, amiRPPO234A and amiRPPO1234 series lines, based on the degree of dark color development that ranged from high to low (Figure 5).

### Statistical correlations

Pearson’s correlation coefficient (r^2^) analysis indicated significantly strong and positive correlations between RPR, RPPO and RBR. (r^2^ = 0.85-0.89, P < 0.0001) in potato tuber tissues (Table 4). Among the potato PPO genes, the *StuPPO2* gene was highly correlated with RPR, RPPO and RBR (r^2^ = 0.70-0.80, P < 0.0001). Both the *StuPPO3* and *StuPPO4* genes were moderately associated with RPR, RPPO and RBR (r^2^ = 0.59-0.71, P < 0.0001), while the *StuPPO1* gene had a weak correlation with the three browning-related parameters, RPR, RPPO and RBR (r^2^ = 0.19-0.27, P < 0.05) (Table 4).

**Table 4:** Pearson correlation coefficients analysis results

	**RPR**	**RPPO**	**RBR**
*StuPPO1*	0.20	0.19	0.27 d
*StuPPO2*	0.70 a	0.78 a	0.80 a
*StuPPO3*	0.59 a	0.69 a	0.71 a
*StuPPO4*	0.66 a	0.69 a	0.65 a
RPR		0.85 a	0.89 a
RPPO			0.87 a

Note: Pearson correlation coefficients (r^2^) of the relationship of PPO gene expression level (*StuPPO1*, *StuPPO2*, *StuPPO3* and *StuPPO4*), relative PPO protein expression level (RPR), relative PPO enzymatic activity (RPPO) and relative browning potential (RBR) in potato tuber tissues. a, P < 0.0001; d, P < 0.05.

**Figure 6:**
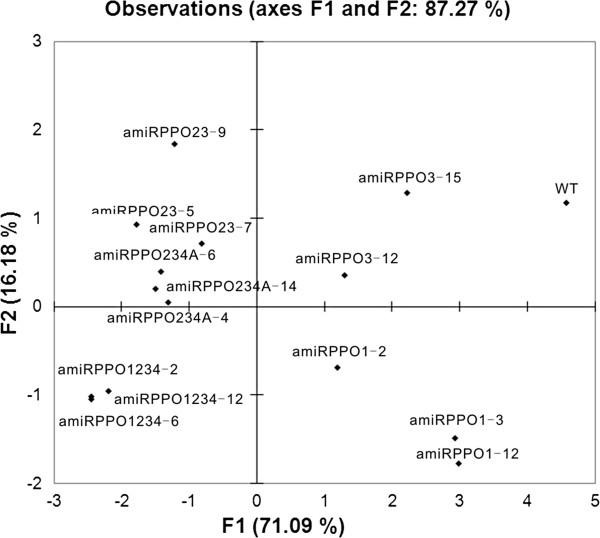
**Score scatter plot of the transgenic lines and WT according to principal component analysis (PCA).** The bioplot represents the first and second principle components (PC1 and PC2, eigenvalue > 1.0) produced from the PCA of 15 observations (14 transgenic lines and WT) based on seven variables, relative PPO gene expression level of *StuPPO1, StuPPO2, StuPPO3 and StuPPO4*, RPR, RPPO and RBR. Each line is represented by one dot. Points that are close together correspond to observations that have similar scores for the components displayed in the plot.

Principal component analysis (PCA) generated only two principle components with eigenvalues exceeding 1.0 (Kaiser’s rule) (Figure 6 and Additional file 5: Table S1). The two components explained 87% of the total variance. The first principle component (PC1) accounted for 71% of total variance and had approximately equal positive loading for the variables *StuPPO2* gene, *StuPPO3* gene, *StuPPO4* gene, RPR, RPPO and RBR. Each of the above variables contributed about 14-18% to the PC1, suggesting their equivalent proportion in the different transgenic lines. In contrast, the variable *StuPPO1* gene also contributed positively to the PC1 but with a lower score (< 0.8% contribution). The second principle component (PC2) only accounted for 16% of the total variance and was mainly influenced by positive loading of *StuPPO1* gene (contribution to PC2, 79%) (Additional file 5: Table S1). The score plot of the PC1 and PC2 paralleled the distribution of the browning phenotypes (Figure 6). Lines amiRPPO1-2, −3 and −12, amiRPPO3-12 and −15 and the WT, susceptible to browning were scattered on the right side of the plot. In contrast, lines amiRPPO23-5, −7 and −9, amiRPPO234A-4, −6 and −14, amiRPPO1234-2, −6 and −12, resistant to browning were separated to the left side of the plot. Noticeably, lines amiRPPO1234-2, −6 and −12, which inhibited *StuPPO1*, *StuPPO2*, *StuPPO3* and *StuPPO4* gene expressions and had the least browning potential, were grouped within the lower quadrant with negative factor scores in both PC1 and PC2, and opposite the WT with positive factor scores in both PC1 and PC2.

**Figure 7:**
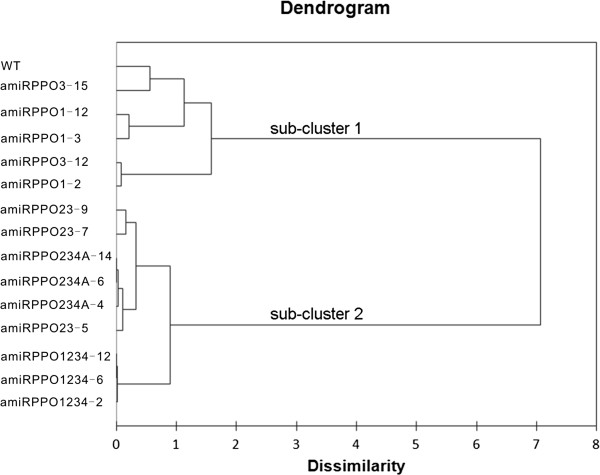
**Dendrogram of hierarchical cluster analysis (HCA) of the transgenic lines and WT.** The dendrogram represents the dissimilarity of the 15 observations (14 transgenic lines and a WT) based on seven variables, relative PPO gene expression level of *StuPPO1, StuPPO2, StuPPO3 and StuPPO4*, RPR, RPPO and RBR according to the HCA (Ward’s linkage hierarchical clustering with the Euclidean distance). On the left of the dendrogram, each observation is considered its own cluster. Horizontal lines represent dissimilarity values, these lines are connected to the lines from other observations with a vertical line. Long horizontal lines represent more distinct separation between the groups. Shorter horizontal lines indicate groups that are not as distinct.

Hierarchical clustering analysis of the transgenic lines on the variables RPR, RPPO, RBR and PPO gene expression levels produced two major clusters, subcluster-1 (top) and subcluster-2 (bottom) (Figure 7). Members in each of the subclusters displayed a similar pattern with regards to expression trends in the variables RPR, RPPO and RBR. Statistically, subcluster-1 expressed considerably higher values (Min, Max and range) of RPR, RPPO and RBR than subcluster-2 (Figure 4). Interestingly, subcluster-1 consisted the WT and the transgenic lines designed for targeting a single PPO gene (*StuPPO1* or *StuPPO3*), including lines amiRPPO1-2, −3 and −12, amiRPPO3-12 and −15, whereas subcluster-2 included transgenic lines designed for targeting multiple PPO genes, namely, the lines amiRPPO23-5, −7 and −9, amiRPPO234A-4, −6 and −14, amiRPPO1234-2, −6 and −12. The transgenic lines were further divided into smaller sub-groups based on their different scores (Figure 7). For example, the group of lines amiRPPO1-3 and amiRPPO1-12 displayed similar level of *StuPPO1* gene suppression but showed almost normal gene transcript levels of *StuPPO2*, *StuPPO3* and *StuPPO4* (Figure 3A). Although the gene transcript levels of both *StuPPO3* and *StuPPO4* in line amiRPPO3-15 were lower by ~70% than those of the WT (Figure 3B), the two clustered together because the values of RPR, RPPO and RBR in amiRPPO3-15 were closer to those in the WT than other transgenic lines (Figure 4A, B and C). Lines amiRPPO1-2 and amiRPPO3-12 grouped based on their similar gene expression levels of *StuPPO2*, *StuPPO3* and *StuPPO4* (Figure 3A and B) and the generally similar values of RPR, RPPO and RBR (Figure 4A, B and C). The group of lines amiRPPO23-7 and amiRPPO23-9 showed strong down-regulation of both the *StuPPO2* and *StuPPO3* genes, moderate down-regulation of the *StuPPO4* gene (Figure 3C), as well as similar RPPO and RBR values (Figure 4B and C). Three amiRPPO234A series lines (−4, −6 and −14) clustered together based on their similar down-regulated gene expression levels for *StuPPO1*, *StuPPO2*, *StuPPO3* and *StuPPO4* (Figures 3D), and their similar low values for RPR, RPPO and RBR (Figure 4A, B and C). The three amiRPPO234A lines further clustered with line amiRPPO23-5, because the four lines performed similarly in almost all of the variables except that the *StuPPO1* mRNA level in the amiRPPO23-5 was moderately higher than in the amiRPPO234A lines (Figures 3C, D, and 4). Three amiRPPO1234 series lines (−2, −6 and −12) grouped closely because they had very similar expression trends in all of the 7 variables (Figures 3E and 4).

## Discussion

PPOs are encoded by a gene family composed of multiple highly conserved gene members in many plant species [2,24]. The differential temporal and spatial expression patterns of PPO genes in potato, poplar and tomato indicate the functional diversities among the PPO gene members [1,33,34]. However, the lack of specific loss-of-function mutants of different PPO genes has impeded progress in understanding the diversified gene functions in the family. Before the present report, several studies reported knockdown of PPO gene expression using siRNA strategies by transformation with hundreds of base pairs of the PPO gene fragments [4-10]. The strategies proved successful in suppression of overall PPO activities and reduction of PPO-mediated browning reactions, but the methods were not able to identify the roles of the individual PPO gene members. The heterogeneous siRNAs generated in the transformed plants did not target a specific PPO gene but affected others of the family as well because the PPO gene family shares high homology in nucleotide sequences among its members [2,24]. Our data demonstrated that PPO gene isoforms can be suppressed individually or simultaneously using amiRNAs. In addition to significantly down-regulating the PPO genes of interest as predicted in our construct design, several exceptions were observed based on the qRT-PCR assay results (Figure 3). For example, an unexpected moderate to high reduction of the *StuPPO2* and *StuPPO3* mRNA levels in line amiRPPO1-2, a *StuPPO1* gene knockdown transformant, and an unforeseen moderate decrease of the *StuPPO1* and *StuPPO2* gene transcript copies in line amiRPPO3-12, a *StuPPO3* gene knockdown mutant were detected although the analogue regions of the affected genes have 6–8 bp mismatches to the targets of the designed amiRNAs (Figure 3A and B, Additional file 1: Part D). An 8-bp complementarity to the 5′ seed region (canonical 8mer site) [35] of the amiRPPO3 in a *StuPPO4* gene sequence region might be related to an unanticipated moderate down-regulation of the non-targeted *StuPPO4* gene in Line amiRPPO3-15 (Figure 3B, Additional file 1: Part D). Apart from strong down-regulations of both *StuPPO2* and *StuPPO3* genes in lines amiRPPO23-5, −7 and −9, *StuPPO4* gene mRNA in these lines was at a moderate to low level, suggesting *StuPPO4* gene expression was also targeted by amiRPPO23 (Figure 3C). The amiRPPO23 was initially designed to target both *StuPPO2* and *StuPPO3* genes based on incomplete *StuPPO4* gene sequence data (GenBank ID: U22923). Later analysis of a *StuPPO4* gene related EST sequence (GenBank ID: BG592710) found a region with only one mismatch to the target of amiRPPO23 (position 11, Additional file 1: Part D). A previous study reported mismatches at position 11 were miRNA admissible targets but reduced miRNA-guided cleaving efficiency *in vitro* and phenotypic effects *in vivo*[36]. Although the possible target region of the *StuPPO1* gene has mismatches at two positions compared to the perfect target sequence of amiRPPO234A (positions 6 and 9 at the amiRPPO234A site, Additional file 1: Part D), the *StuPPO1* gene was moderately down-regulated in amiRPPO234A-4, −6 and −14 lines, along with strong down-regulation of the *StuPPO2*, *StuPPO3* and *StuPPO4* genes (Figure 3D). It is worth noting that G:U wobble pairing was introduced for targeting *StuPPO3* gene in amiRPPO234A and *StuPPO1* gene in amiRPPO1234 (Table 2) [37], and the corresponding target genes, *StuPPO3* gene in the amiRPPO234A series lines and *StuPPO1* gene in the amiRPPO1234 series lines, were indeed down-regulated in both cases (Figure 3D and E). We failed to detect the designed amiRNAs from any of the transgenic plants transformed with the constructs for amiRPPO2 (transgenic amiRPPO2 series lines) and amiRPPO234 (transgenic amiRPPO234 series lines) for unknown reasons (Table 3). Investigation of the principles and complexities between amiRNAs and target recognition was beyond the scope of this study. Nonetheless, our data showed that amiRNAs are a potent regulator for modulating expression of potato PPO gene isoforms individually or in combination, and amiRNA targets are more predictable and detectable than the siRNA’s. However, not all designed amiRNAs were expressed with similar efficiency in the transgenic plants and off-target effects also occurred occasionally. Appropriate detection methods are required for screening the desired transgenic traits in plants expressing amiRNAs.

Individual or cumulative down-regulation of PPO genes did not usually cause up-regulation of the other PPO genes, except for transgenic line amiRPPO1-12 that showed a reduction of the targeted *StuPPO1* gene mRNA level by about 90% but also an increase of approximately 40% of the non-targeted *StuPPO2* gene transcript (Figure 3A). This implied that different PPO genes may be regulated independently reflecting their diversified functions in the potato plant. Suppression of PPO gene expressions in our transgenic plants resulted in a reduced level of total PPO protein, but the reductions varied depending on the knockdown targets. For example, expression of *StuPPO1* was suppressed by 90 to 98% in lines amiRPPO1-3 and amiRPPO1-12 (Figure 3A), and the total PPO protein level in these two transgenic lines was reduced by ~20 to 30% (Figure 4A). Coincidentally, the transgenic lines amiRPPO23-5, −7 and −9, which simultaneously down-regulated the *StuPPO2*, *StuPPO3* and *StuPPO4* gene (Figure 3C), had reduced total PPO protein level of about 70% (Figure 4A). Total PPO protein level was reduced by 15% on average in line amiRPPO3-15 that showed suppression of the *StuPPO3* gene (target) and *StuPPO4* gene (off-target) (Figures 3B and 4A). These data indicated that *StuPPO2* protein probably contributed 55% or more to the total PPO protein levels in the non-transgenic potato tuber tissues. The failure to generate *StuPPO2*-knockdown mutants made it difficult to evaluate the accuracy of the estimation. However, Pearson correlation coefficient indicated that 70% of the variation in total PPO protein level in potato tuber tissue was explained by *StuPPO2* gene expression (r^2^ = 0.70, Table 3). Analysis of PPO gene expression in the non-transgenic potato tubers by qRT-PCR found that the *StuPPO2* gene contributed 67% to the total PPO gene transcript level, followed by *StuPPO1*, 28%, *StuPPO3*, 4% and *StuPPO4*, less than 0.2% (Additional file 6: Table S2). This also indicated that *StuPPO2* is a major contributor to the PPO protein content of the WT potato tubers. Noticeably, *StuPPO1*, *StuPPO2*, *StuPPO3* and *StuPPO4* genes were all down-regulated by 90% or more in transgenic amiRPPO1234-2, −6 and −12 lines (Figure 3E), but the total PPO protein level remained between 20 and 30% in their tuber tissues. An explanation for this is that the PPO proteins that accumulate in the plastid probably accumulated for a longer duration than the cytosol-produced PPO mRNAs [1,2].

A striking reduction of PPO-mediated browning has been described previously through suppression of overall PPO gene expression using siRNA strategies [4-6]. Here, we have shown that it requires that *StuPPO1*, *StuPPO2*, *StuPPO3* and *StuPPO4* genes were all suppressed simultaneously to achieve optimal inhibition of PPO activity and non-browning phenotype in transgenic potato tuber tissue (Figures 4 and 5), a point not well known or understood previously. It was of particular interest that a series of low browning phenotypes were generated though suppression of the highly conserved PPO gene isoforms when suppressed both individually and in combination using this amiRNA strategy (Figure 5). Analysis of these transgenic clones clearly demonstrated the positive correlations between PPO protein levels (RPR), PPO enzymatic activity (RPPO) and browning potential (RBR) (Figure 4, Table 4). For example, a ~20-30% reduction at PPO protein level in the tubers of lines amiRPPO1-3 and −12, where the *StuPPO1* gene expression were down-regulated by over 90%, showed a parallel decrease of both PPO activity and browning potential in the tuber tissues. Simultaneous down-regulation of *StuPPO1*, *StuPPO2*, *StuPPO3* and *StuPPO4* genes in lines amiRPPO1234-2, −6 and −12 resulted in 70-80% loss of tuber PPO protein. As a result of this, PPO enzymatic activity was reduced by ~90% and browning potential by 70-75%. Browning potential correlated better with the PPO protein level than with PPO enzymatic activity. PPO enzymatic activity appeared to decrease relatively faster in our assays than browning reaction (Figure 4). The difference may have resulted from different sample preparation methods used for the two assays. PPO enzymatic activity was more temporally sensitive after the enzymes were mechanically isolated from the plastids, while browning potential recorded the accumulation of pigments in the samples during the time period of the assay. In spite of the small discrepancy between these two data sets, the trend was consistent among the PPO gene down-regulated transgenic plants; the lower the PPO protein level in the potato tuber tissues, the less the PPO activity and browning potential.

Our survey of the recent *S. tuberosum* whole genome database (http://www.jgi.doe.gov) revealed 9 PPO-like gene models (Table 1, Additional file 1). *StuPPO1* to *StuPPO8* are clustered on chromosome 8 and only *StuPPO9* is located on chromosome 2. *StuPPO1*, *StuPPO2*, *StuPPO3* and *StuPPO4* are allelic to previously characterized *POTP1/P2*, *POT32*, *POT33* and *POT72*, respectively, in this report, based on their 95-99% identities at nucleotide level [1,12]. The deduced peptides also show 95-98% identical positions between the corresponding alleles (Additional file 1). Surprisingly, *StuPPO1* is the only possible allele to the *POTP1* and the *POTP2* genes, and no duplication of the *StuPPO1* locus in the *S. tuberosum* genome was discovered. *StuPPO5* to *StuPPO9* are five novel PPO-like gene models predicted in the *S. tuberosum* genome (Table 1, Additional file 1). However, the low to no prevalence in the EST databases for the *StuPPO5*, *StuPPO6*, *StuPPO7* and *StuPPO8* implies these gene models may be under-transcribed in *S. tuberosum* (Additional file 1: Part C). In addition, the important tyrosinase domains (pfam00264) for PPO enzymatic activity are incomplete in the peptides of the deduced translation of the *StuPPO5* and *StuPPO7* gene models, suggesting that these two may not be functional like other PPO isoforms (Additional file 1: Part B) [23,24]. *StuPPO9* is probably a PPO-like gene involved in disease defense and cell rescue because the ESTs that support for this gene model were dominantly discovered in the tissues of *in vitro* cultured potato callus, abiotic stress treated leaf and root or pathogen-infected leaf and tuber, and several of the ESTs expressed at significantly higher levels in the pathogen-infected potato tissues (Additional file 1: Part C) [25-28]. Taken together, the number of members of the potato PPO gene family are possibly larger than previously reported, but *StuPPO1* (*POTP1/P2*), *StuPPO2* (*POT32*), *StuPPO3* (*POT33*) and *StuPPO4* (*POT72*) are probably the major developmentally regulated PPO genes in *S. tuberosum* and they are subjected to the targets of the amiRNAs expressed in the transgenic lines. However, extensive experimental studies are required to investigate the existence and function of the potential *StuPPO5 to StuPPO9* genes.

## Conclusions

PPO-mediated browning damage is one of the main causes of quality loss in fresh fruit and processed food. It is of great importance and interest to produce crop varieties with low PPO activity for the food industry. Our results have shown that *StuPPO1* (*POTP1/P2*), *StuPPO2* (*POT32*), *StuPPO3* (*POT33*) and *StuPPO4* (*POT72*) genes were the major contributors to the total PPO protein content but the effect of the individuals was not equal and that PPO activity in the tuber tissues paralleled the protein content data. Suppression of expression of one or a few PPO genes did not cause overexpression of the others, but the greatest reduction of PPO activity and the most complete non-browning phenotypes required simultaneous suppression of the expression of *StuPPO1*, *StuPPO2*, *StuPPO3* and *StuPPO4* genes. Our demonstration that PPO gene expression in potato can be suppressed by introduction of 21-nt small RNA regulators provides a new strategy for developing low- or non-browning crops. It also suggests that amiRNAs can be applied to silence closely related members of multi-gene family for functional genomics study in non-model plants. In addition, a series of PPO gene knockdown mutants predictably generated provide us important resources for future investigations of the role of PPO genes in functions such as plant development, stress and insect and fungal defense response.

## Methods

### Survey of PPO-like genes in *Solanum tuberosum*

Nucleotide sequence information of the identified potato PPO genes, including *POTP1* (GenBank ID: M95196), *POTP2* (M95197), *POT32* (U22921), *POT33* (U22922), and *POT72* (U22923) were obtained from the GenBank. The gene sequences were used as queries to search for the PPO-like gene sequences from a recent *S. tuberosum* whole genome database in the US Joint Genome Institute (http://www.jgi.doe.gov) using the blastn with the default parameters. The BLAST hits were manually checked and the PPO-like gene sequences were retrieved and further analyzed using Vector NTI Advance 11 software (Life Technology, USA), NCBI BLAST platform (blastn, blastp, nr/nt, ESTs, etc.), and Simple Modular Architecture Research Tool (SMART, http://smart.embl-heidelberge.de/).

### Construction of amiRNA vectors

The amiRNAs for targeting the characterized potato PPO genes, including *StuPPO1* (previously named *POTP1/P2*), *StuPPO2* (previously, *POT32*), *StuPPO3* (previously, *POT33*) and *StuPPO4* (previously, *POT72*), individually or cumulatively were designed in an *Arabidopsis* miR-168a gene backbone using an in-house amiRNA designer and constructed as previously described [30]. All constructs were sequenced to confirm the intended construction and the designs. Descriptions of the amiRNAs, their target sequences and genes are listed in Table 2.

### Plant genetic transformation

Transformation of amiRNA constructs with potato cultivar ALT1762 was performed as previously described [30]. Transgenic plants with each of the amiRNA designs were assigned a series of names and numbers as listed in Table 3.

### Analysis of PPO gene expression by real-time quantitative reverse transcription PCR (qRT-PCR)

Potato tissues were disrupted in liquid nitrogen using mortar and pestle. Total RNA was extracted using a Spectrum plant total RNA kit (Sigma-Aldrich, USA) according to the manufacturer’s instruction. The purified total RNA samples were treated with Ambion TURBO DNase (Life Technologies, USA) to eliminate potential contamination from genomic DNA, and subsequently re-purified using RNeasy mini kit (Qiagen, Germany). RNA concentration and purity were determined using a ND-1000 spectrophotometer (NanoDrop, Wilmington, DE, USA). First strand cDNA was synthesized from 1 μg of the re-purified total RNA using 1 μl of RT primer mix (provided with the QuantiTect reverse transcription kit, Cat. No. 205310, Qiagen) and 1 μl of SuperScript III reverse transcriptase (200 U/μl) in a 20-μl reaction according to the manufacturer’s instruction (Life technologies).

Two housekeeping genes, translation elongation factor 1-alpha (ef1α) and cyclophilin were selected as references for qRT-PCR data analysis. The primer pairs for ef1α and cyclophilin gene amplicons were the same as the designs by Nicot et al. [38] (Additional file 7: Table S3). The expression stability of the two reference genes in potato tuber tissues was validated by geNorm algorithm [39] (Additional file 8: Figure S4). More than 30 primers for amplions of PPO genes were designed using the Primer 3 (version 0.4.0) [40] based on available gene–specific nucleotide sequence information retrieved from the GenBank databases. Four primer pairs, one for each of the PPO genes, namely *StuPPO1*, *StuPPO2*, *StuPPO3* and *StuPPO4* were eventually selected for qRT-PCR analysis in this study. The PCR amplification specificities using these primer pairs were examined by agarose gel electrophoresis, cloning and sequencing, as well as post-PCR melting curve analysis (Additional file 7: Table S3). All primers used in this research were synthesized by the Integrated DNA Technologies, Inc. (Iowa, USA).

All qRT-PCRs were carried out in triplicate in 96-well microplates and performed in a CFX96 real-time PCR detection system (Bio-Rad, USA). Each reaction volume was 20 μl, comprised of 10 μl of Eva green master mix (Applied Biological Materials Inc., Canada), 0.5 μM of forward and reverse primers (primer pair mix, 5 μl) and 5 μl of appropriately diluted DNA or cDNA template. No-template control (NTC) reactions were included in each plate to monitor potential formation of primer-dimers. The thermal cycling was programed as follows: one initial denaturation cycle at 95°C for 10 min; 40 cycles at 95°C for 30 s and 60°C for 30 s. Fluorescence signal was measured at the end of each annealing and extension step at 60°C. At the end of the qRT-PCR run, a melting curve analysis with a temperature gradient of 0.1°C/s from 65 to 95°C was performed to ensure that only single products were produced. Relative expression levels of the PPO gene expressions were calculated based on a method described in Pfaffl [41]. Data normalization was performed using the gene expression values of the ef1α and cyclophilin in the samples.

### Detection of amiRNAs by reverse transcription PCR (RT-PCR)

Detections of mature amiRNAs expressed in the transgenic plants by RT-PCR were conducted as previously described [30]. The forward primers used in the PCR were identical to the corresponding amiRNA sequences except that they were synthesized as oligodeoxynucleotides (Additional file 9: Table S4).

### Detection of cleaved mRNA of amiRNA target

The FirstChoice RLM-RACE kit (Life Technologies) was explored to detect the cleaved mRNA product of the amiRNA target. The kit is designed to amplify cDNA only from full-length, capped mRNA by removing free 5′-phophates from molecules such as fragmented mRNA using Calf-intestinal alkaline phosphatase (CIAP) before processing to remove the cap structure from full-length mRNA using Tobacco acid pyrophosphatase (TAP). In our application, the CIAP and TAP treatment steps were omitted to prevent full-length, capped-mRNA from ligating to the 5′-RACE adapter (Figure 2A).

Poly(A)^+^ RNA was purified using the Dynabead mRNA Purification kit (Life Technologies). Enriched poly(A)^+^ RNA was ligated to the 5′-RACE adapter provided in the FirstChoice kit without treatment with CIAP and TAP for exclusion of all 5′-capped mRNA. The conditions for 1st-strand cDNA synthesis, the followed-up PCR and nested PCR were carried out as instructed by the manufacturer. Besides that, the following primer strategy was developed for detecting possible fragmented mRNA from *StuPPO1*, *StuPPO2*, *StuPPO3* and *StuPPO4* genes (Figure 2A). A reverse primer designed from a conserved sequence region of all four PPO genes was used to synthesize the 1st-strand cDNA. Two other reverse primers designed from another two conserved regions of the four PPO genes were used with the common 5′-RACE primer to conduct the 1st-round PCR and the follow-up nested-PCR-1. Using products from the nested-PCR-1 as the template, nested-PCR-2 reactions were performed with the common 5′-RACE primer and four gene-specific reverse primers, respectively for detecting the bio-origination of the cleaved mRNA products. Distinct bands of appropriate size of PCR fragments were separated by electrophoresis, cloned and sequenced. The oligonucleotides used for detection of cleaved mRNA are listed in Additional file 10: Table S5.

### Analysis of PPO protein levels in potato tuber tissues

A protein dot blot assay was used for semi-quantifying PPO protein levels in potato tubers. Crude protein samples were prepared by powdering 0.2 g tuber tissue in liquid nitrogen followed by adding 1.6 ml of extraction buffer (2% SDS, 0.1% β-mercaptoethanol in 63 mM Tris–HCl buffer, pH 6.8). Samples were boiled for 10 min, followed by centrifugation at 20,000 *g* for 2 min to remove insoluble material. The crude protein concentrations were determined using the BCA Protein Assay Kit (Thermo Scientific Pierce, USA) and all samples were then adjusted to 100 mg/ml protein stock. For dot blot assay, 1:400 diluted samples from the protein stocks were blotted onto polyvinylidene difluoride membrane using Easy-Titer ELIFA System 77000 (Pierce) and probed with a PPO specific antibody (Ab). The PPO Ab was raised in rabbit against a synthetic peptide (KDWLNSEFFFYDE) corresponding to a conserved region in the deduced StuPPO1, StuPPO2, StuPPO3 and StuPPO4 protein sequences by Applied Biological Materials Inc. (Canada). Two-fold serial dilutions of the synthetic peptide (KDWLNSEFFFYDE) from 50 ng to 0.78125 ng were included in each blot for internal control and making protein standards. The PPO Ab probed blot was incubated with HRP-conjugated anti-rabbit IgG and detected with the Enhanced Chemiluminescence Detection System (GE-Amersham, USA). PPO Protein concentrations were determined using Aida Image Analyzer version 2.00. Relative protein expression level, i.e. PPO proteins in the transgenic plants to that in wild type was calculated for presentation in this study.

### PPO enzymatic activity assay

PPO enzymatic activity was measured using 4-methylcatechol (4-MC) as substrate. A 40 mg sample with a dimension of 2 mm × 5 mm × 5 mm was collected from the potato tuber tissue next to the skin using a fruit peeler and a single-hole paper punch, and frozen in liquid nitrogen immediately. The sample was homogenized with cold PPO extraction buffer (100 mM sodium phosphate buffer pH 6.0, 2% TX-100, 2% PVPP) using a Fast Prep FP120A-115 Homogenizer (Thermo Electron Corp, USA). A ten-fold diluted extract (20 μl) was mixed with 200 μl of the assay mixture (50 mM sodium phosphate buffer pH 6.0, 0.1% SDS and 15 mM 4-MC) in 96-well Microplates. A SpectraMax M2 Microplate Reader (Molecular Devices, USA) was used to measure the absorbance increase at 400 nm (*A*_400nm_) every 5 seconds for 1 min at 25°C. One unit of PPO enzymatic activity was defined as the amount of enzyme necessary to change *A*_400nm_ in 0.001/min at 25°C. The total protein concentration of the extract was detected using the BCA Protein Assay Kit (Pierce) and measured in the same SpectraMax M2 Microplate Reader. The enzymatic activity was calculated as U/mg of total protein.

### Browning potential assay

Browning potential of phenolic compounds in potato tuber tissues was measured using a modification of the method described previously by [32]. Each sample tissue was a collection of two randomly selected potato tubers harvested from the same transgenic line or wild type. Triple biological replicates (representing a total of 6 tubers per line) were set up for each treatment. The skins were peeled off before the tubers were chopped into small pieces manually. A 3-gram subsample of each pooled two-tuber replicate was weighed out and homogenized with 15 ml cold sodium phosphate buffer (50 mM, pH 6.0) using an Ultra-Turrax tissue homogenizer (Takmar, Cincinnati, Ohio) at high speed for 1 min. The homogenate was allowed to oxidize at room temperature for 1 h before it was vortexed and 1 ml aliquots were centrifuged at 12,000 *g* for 10 min. 300 μl of the supernatant was loaded in a 96-well microplate for measuring the absorbance at 475 nm using a SpectraMax M2 Microplate Reader (Molecular Devices) and the A_475nm_ value was used as an indication of the browning potential.

### Statistics analysis

Analysis of variance with the general linear model procedures, means comparison by Duncan’s multiple range test and the Pearson’s correlation analysis were performed according to Statistical Analysis System, SAS 9.1 for windows (SAS Institute, Cary, NC). Principal component analysis (PCA) was conducted with XLSTAT 2012 for Windows (Addinsoft, NY). Hierarchical cluster analysis (HCA) was carried out with SYSTAT 12 version 12.02 for Windows (Addinsoft, NY) using Ward’s linkage hierarchical clustering with the Euclidean distance.

## Abbreviations

amiRNA: Artificial microRNA; EST: Expressed sequence tag; PPO: Polyphenol oxidase; qRT-PCR: Real-time quantitative reverse transcription PCR; RACE-PCR: Rapid amplification of cDNA ends PCR; RT-PCR: Reverse transcription PCR; RBR: Relative browning potential; RPPO: Relative PPO activity; RPR: Relative PPO protein level; StuPPO: *Solanum tuberosum* polyphenol oxidase; WT: Wild type.

## Competing interests

The authors declare that they have no competing interests.

## Authors’ contributions

YX, WDL, GT, MC, YS, and RS conceived and designed the research. GT designed miRNA constructs. MC, BB, and YX designed and performed the experiments. MC, YX, BB, GT, BDO and PAW analyzed the data. YX, MC, BB, and WDL wrote the article. GT, BDO, YS, and RS revised the article. All authors read and approved the final manuscript.

## Supplementary Material

Additional file 1 **Genome-wide analysis of the PPO gene family in *Solanum tuberosum*.** Part A, PPO-like genes predicated based on the *S. tuberosum* genome database; Part B, Confidently predicted domains in the deduced StuPPO1 to StuPPO9 protein sequences using SMART; Part C, List of the supporting ESTs and their biological sources. Part D, List of the amiRNA name, amiRNA sequence, amiRNA target sequence and the target homologous sequences of 9 PPO-like gene models. Click here for file

Additional file 2: Figure S1 Detection of amiRNAs expressed in transgenic potatoes by reverse transcription PCR (RT-PCR). Click here for file

Additional file 3: Figure S2 Sequence alignment of the clones of the 5′ RACE-PCR product from transgenic line amiRPPO1-12. Click here for file

Additional file 4: Figure S3 Sequence alignment of the cDNA of the truncated PPO mRNA product from transgenic line amiRPPO1-12 with *StuPPO1/POTP1/POTP2*. Click here for file

Additional file 5: Table S1 Results of principal component analysis (PCA) of the transgenic lines and WT. Click here for file

Additional file 6: Table S2 Relative expression level of PPO genes in the tuber tissue of potato cultivar Alt-1762 (wild type). Click here for file

Additional file 7: Table S3 Primers and amplicon characteristics of reference genes and PPO genes used in quantification of PPO gene expression in potato tuber tissue measured by qRT-PCR. Click here for file

Additional file 8: Figure S4 Stability test of candidate reference genes in potato tuber tissue. Click here for file

Additional file 9: Table S4 List of forward primers used for detection of amiRNAs by reverse transcription PCR. Click here for file

Additional file 10: Table S5 List of reverse primers used in detection of the cleaved mRNA of amiRNA target in transgenic lines amiRPPO1-12 by 5′-RACE PCR. Click here for file
