# Supplementary figures and images for: Reduced polyphenol oxidase gene expression and enzymatic browning in potato (Solanum tuberosum L.) with artificial microRNAs
Source: BMC Plant Biol. 2014 Mar 11;14:62. doi: 10.1186/1471-2229-14-62 (PMC4007649; doi:10.1186/1471-2229-14-62)

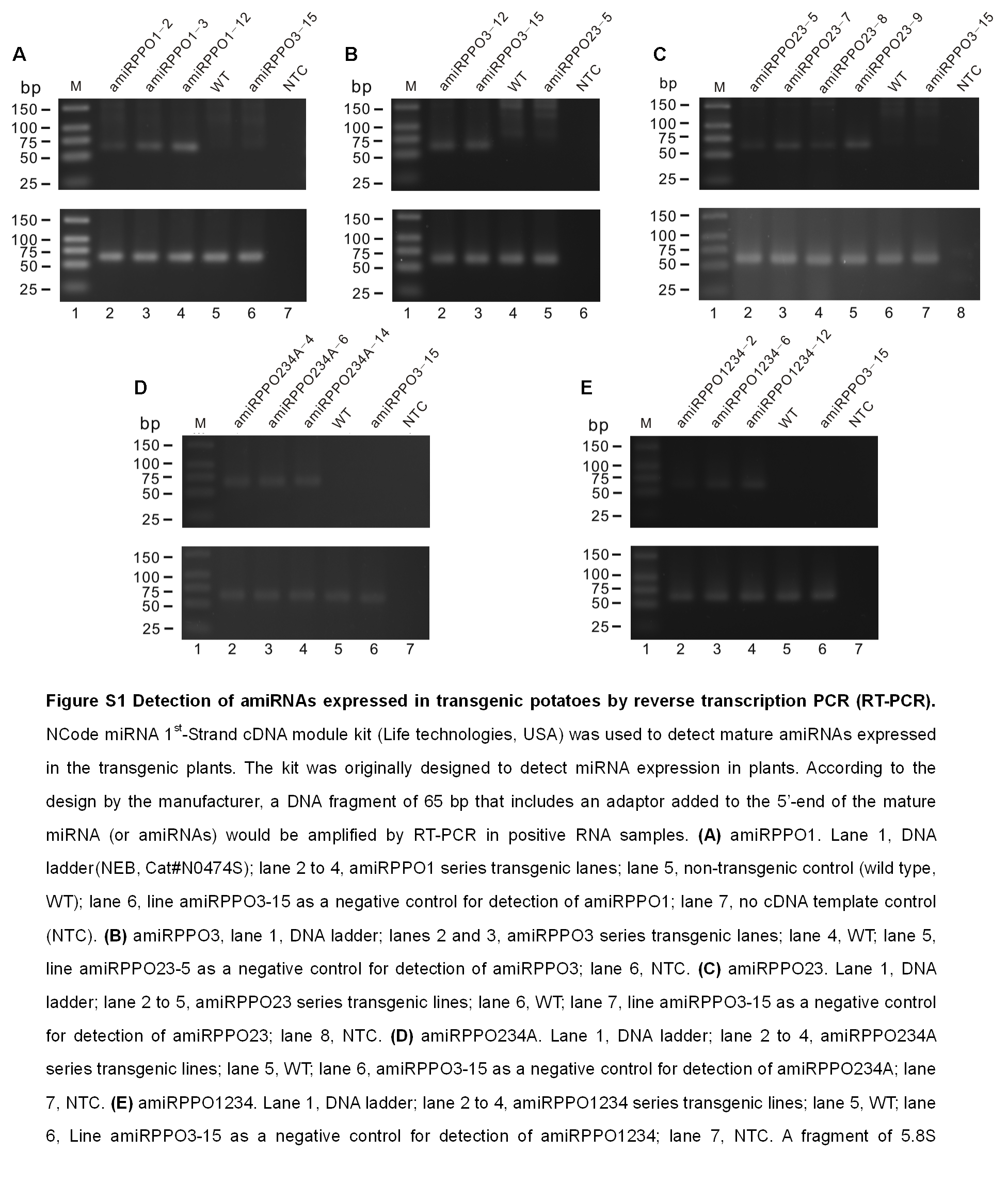

Supplement: Additional file 2: Figure S1 — Detection of amiRNAs expressed in transgenic potatoes by reverse transcription PCR (RT-PCR). [file 1471-2229-14-62-S2.tiff]

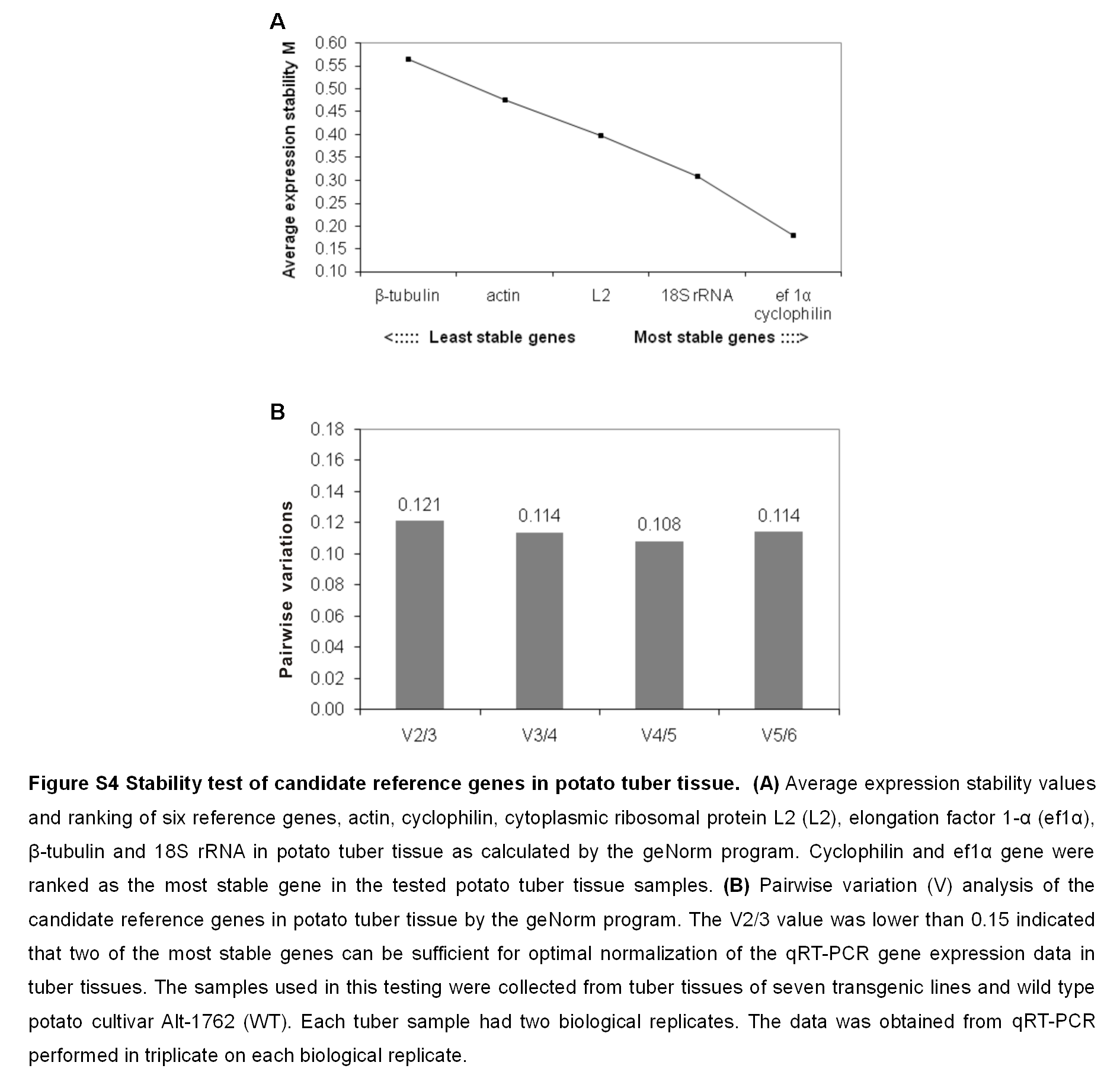

Supplement: Additional file 8: Figure S4 — Stability test of candidate reference genes in potato tuber tissue. [file 1471-2229-14-62-S8.tif]
