# Supplementary material for: Reduced polyphenol oxidase gene expression and enzymatic browning in potato (Solanum tuberosum L.) with artificial microRNAs
Source: BMC Plant Biol. 2014 Mar 11;14:62. doi: 10.1186/1471-2229-14-62 (PMC4007649; doi:10.1186/1471-2229-14-62)
Supplement: Additional file 3: Figure S2 — Sequence alignment of the clones of the 5′ RACE-PCR product from transgenic line amiRPPO1-12. [file 1471-2229-14-62-S3.docx]

1 50

R2 (1) GCTGATGGCGATGAATGAACACTGCGTTTGCTGGCTTTGATGAAACAGTC

R3 (1) GCTGATGGCGATGAATGAACACTGCGTTTGCTGGCTTTGATGAAACAGTC

R4 (1) GCTGATGGCGATGAATGAACACTGCGTTTGCTGGCTTTGATGAAACAGTC

R5 (1) GCTGATGGCGATGAATGAACACTGCGTTTGCTGGCTTTGATGAAACAGTC

R6 (1) GCTGATGGCGATGAATGAACACTGCGTTTGCTGGCTTTGATGAAACAGTC

R8 (1) GCTGATGGCGATGAATGAACACTGCGTTTGCTGGCTTTGATGAAACAGTC

R10 (1) GCTGATGGCGATGAATGAACACTGCGTTTGCTGGCTTTGATGAAACAGTC

Consensus (1) GCTGATGGCGATGAATGAACACTGCGTTTGCTGGCTTTGATGAAACAGTC

51 100

R2 (51) ACCAAGGTGTTCCCACTAGCGAAGCTGGACCGTGCGATTTCGTTCTCTAT

R3 (51) ACCAAGGTGTTCCCACTAGCGAAGCTGGACCGTGCGATTTCGTTCTCTAT

R4 (51) ACCAAGGTGTTCCCACTAGCGAAGCTGGACCGTGCGATTTCGTTCTCTAT

R5 (51) ACCAAGGTGTTCCCACTAGCGAAGCTGGACCGTGCGATTTCGTTCTCTAT

R6 (51) ACCAAGGTGTTCCCACTAGCGAAGCTGGACCGTGCGATTTCGTTCTCTAT

R8 (51) ACCAAGGTGTTCCCACTAGCGAAGCTGGACCGTGCGATTTCGTTCTCTAT

R10 (51) ACCAAGGTGTTCCCACTAGCGAAGCTGGACCGTGCGATTTCGTTCTCTAT

Consensus (51) ACCAAGGTGTTCCCACTAGCGAAGCTGGACCGTGCGATTTCGTTCTCTAT

101 150

R2 (101) CACCAGACCAGCTTCGTCAAGGACTACACAGGAGAAAAATGAGCAAGAGG

R3 (101) CACCAGACCAGCTTCGTCAAGGACTACACAGGAGAAAAATGAGCAAGAGG

R4 (101) CACCAGACCAGCTTCGTCAAGGACTACACAGGAGAAAAATGAGCAAGAGG

R5 (101) CACCAGACCAGCTTCGCCAAGGACTACACAGGAGAAAAATGAGCAAGAGG

R6 (101) CACCAGACCAGCTTCGTCAAGGACTACACAGGAGAAAAATGAGCAAGAGG

R8 (101) CACCAGACCAGCTTCGTCAAGGACTACACAGGAGAAAAATGAGCAAGAGG

R10 (101) CACCAGACCAGCTTCGTCAAGGACTACACAGGAGAAAAATGAGCAAGAGG

Consensus (101) CACCAGACCAGCTTCGTCAAGGACTACACAGGAGAAAAATGAGCAAGAGG

151 200

R2 (151) AGATACTGACATTCAACAAAATAGCCTATGATGATACTCAGTATGTAAGG

R3 (151) AGATACTGACATTCAACAAAATAGCCTATGATGATACTCAGTATGTAAGG

R4 (151) AGATACTGACATTCAACAAAATAGCCTATGATGATACTCAGTATGTAAGG

R5 (151) AGATACTGACATTCAACAAAATAGCCTATGATGATACTCAGTATGTAAGG

R6 (151) AGATACTGACATTCAACAAAATAGCCTATGATGATACTCAGTATGTAAGG

R8 (151) AGATACTGACATTCAACAAAATAGCCTATGATGATACTCAGTATGTAAGG

R10 (151) AGATACTGACATTCAACAAAATAGCCTATGATGATACTCAGTATGTAAGG

Consensus (151) AGATACTGACATTCAACAAAATAGCCTATGATGATACTCAGTATGTAAGG

201 250

R2 (201) TTCGATGTGTTCCTGAACGTTGACAAGACTTTGAATGCGAATGAGCTTGA

R3 (201) TTCGATGTGTTCCTGAACGTTGACAAGACTTTGAATGCGAATGAGCTTGA

R4 (201) TTCGATGTGTTCCTGAACGTTGACAAGACTGTGAATGCGGATGAGCTTGA

R5 (201) TTCGATGTGTTCCTGAACGTTGACAAGACTGTGAATGCGGATGAGCTTGA

R6 (201) TTCGATGTGTTCCTGAACGTTGACAAGACTGTGAATGCGGATGAGCTTGA

R8 (201) TTCGATGTGTTCCTGAACGTTGACAAGACTGTGAATGCGAATGAGCTTGA

R10 (201) TTCGATGTGTTCCTGAACGTTGACAAGACTGTGAATGCGGATGAGCTTGA

Consensus (201) TTCGATGTGTTCCTGAACGTTGACAAGACTGTGAATGCGGATGAGCTTGA

251

R2 (251) CAA

R3 (251) CAA

R4 (251) CAA

R5 (251) CAA

R6 (251) CAA

R8 (251) TAA

R10 (251) CAA

Consensus (251) CAA

**Figure S2 Sequence alignment of the clones of the 5’ RACE-PCR product from transgenic line amiRPPPO1-12.** The nested PCR-1 products were 253bp in length, including a 45 bp of 5’ RACE Adaptor and a 24 bp of reverse primer (oligo 10, Additional file 10, Table S5) region (both are underlined at the consensus row). The aligned sequences showed only one nucleotide difference among the seven individual clones ignoring the degenerated oligo 10 region
