# Supplementary material for: Reduced polyphenol oxidase gene expression and enzymatic browning in potato (Solanum tuberosum L.) with artificial microRNAs
Source: BMC Plant Biol. 2014 Mar 11;14:62. doi: 10.1186/1471-2229-14-62 (PMC4007649; doi:10.1186/1471-2229-14-62)
Supplement: Additional file 4: Figure S3 — Sequence alignment of the cDNA of the truncated PPO mRNA product from transgenic line amiRPPO1-12 with StuPPO1/POTP1/POTP2. [file 1471-2229-14-62-S4.docx]

1 50

*POTP1* (M95196) (1) CAGTCACCAAGGTGTTCCCACTGGCGAAGCTGGACCGTGCGATTTCATTC

*POTP2* (M95197) (1) CAGTCACCAAGGTGTTCCCACTAGCGAAGCTGGACCGTGCAATTTCGTTC

*StuPPO1* (MX_006355177) (1) CAGTCACCAAGGTGTTCCCACTAGCGAAGCTGGACCGTGCGATTTCGTTC

R5 (1) CAGTCACCAAGGTGTTCCCACTAGCGAAGCTGGACCGTGCGATTTCGTTC

R10 (1) CAGTCACCAAGGTGTTCCCACTAGCGAAGCTGGACCGTGCGATTTCGTTC

Consensus (1) CAGTCACCAAGGTGTTCCCACTAGCGAAGCTGGACCGTGCGATTTCGTTC

51 100

*POTP1* (M95196) (51) TCTATCACCAGACCAGCTTCGTCAAGGACTACGCAGGAGAAAAATGAGCA

*POTP2* (M95197) (51) TCTATCACCAGACCAGCTTCGTCAAGGACTACACAGGAGAAAAATGAGCA

*StuPPO1* (MX_006355177) (51) TCTATCACCAGACCAGCTTCGTCAAGGACTACACAGGAGAAAAATGAGCA

R5 (51) TCTATCACCAGACCAGCTTCGCCAAGGACTACACAGGAGAAAAATGAGCA

R10 (51) TCTATCACCAGACCAGCTTCGTCAAGGACTACACAGGAGAAAAATGAGCA

Consensus (51) TCTATCACCAGACCAGCTTCGTCAAGGACTACACAGGAGAAAAATGAGCA

101 150

*POTP1* (M95196) (101) AGAGGAGATACTGACATTCAAAAAGATAGCCTATGATGATACTCAGTATG

*POTP2* (M95197) (101) AGAGGAGATACTGACATTCAACAAAGTAGCCTATGATGATACTAAGTATG

*StuPPO1*-MX_006355177 (101) AGAGGAGATACTGACATTCAACAAAATAGCCTATGATGATACTCAGTATG

R5 (101) AGAGGAGATACTGACATTCAACAAAATAGCCTATGATGATACTCAGTATG

R10 (101) AGAGGAGATACTGACATTCAACAAAATAGCCTATGATGATACTCAGTATG

Consensus (101) AGAGGAGATACTGACATTCAACAAAATAGCCTATGATGATACTCAGTATG

151 184

*POTP1* (M95196) (151) TAAGGTTCGATGTGTTCCTGAACGTTGACAAGAC

*POTP2* (M95197) (151) TAAGGTTCGATGTGTTCCTGAACGTTGACAAGAC

*StuPPO1* (MX_006355177) (151) TAAGGTTCGATGTGTTCCTGAACGTTGACAAGAC

R5 (151) TAAGGTTCGATGTGTTCCTGAACGTTGACAAGAC

R10 (151) TAAGGTTCGATGTGTTCCTGAACGTTGACAAGAC

Consensus (151) TAAGGTTCGATGTGTTCCTGAACGTTGACAAGAC

**Figure S3 Sequence alignment of the cDNA of the truncated PPO mRNA product from transgenic line amiRPPO1-12 with the *StuPPO1* gene (GenBank accession number: M95196 / M95197 / MX_006355177).** The 184 bp of the 5’ RACE-PCR products (R5 and R10), which excluded the sequences of the 5’ RACE Adaptor and 3’ end reverse primer region, are aligned with *POTP1* (M95196: nucleotide position 1333-1516), *POTP2* (M95197: nucleotide position 1347-1530) and *StuPPO1* gene (MX_006355177: nucleotide position 1358-1541).
