# Supplementary material for: Reduced polyphenol oxidase gene expression and enzymatic browning in potato (Solanum tuberosum L.) with artificial microRNAs
Source: BMC Plant Biol. 2014 Mar 11;14:62. doi: 10.1186/1471-2229-14-62 (PMC4007649; doi:10.1186/1471-2229-14-62)
Supplement: Additional file 5: Table S1 — Results of principal component analysis (PCA) of the transgenic lines and WT. [file 1471-2229-14-62-S5.docx]

**Table S1 Results of principal component analysis (PCA) of the transgenic lines and WT**

**(a)** Eigenvalues, variability (%) and cumulative (%) of principle components

|  | F1 | F2 | F3 | F4 | F5 | F6 | F7 |
| --- | --- | --- | --- | --- | --- | --- | --- |
| Eigenvalue | 4.976 | 1.132 | 0.408 | 0.306 | 0.096 | 0.065 | 0.017 |
| Variability (%) | 71.088 | 16.177 | 5.830 | 4.367 | 1.367 | 0.933 | 0.237 |
| Cumulative % | 71.088 | 87.266 | 93.095 | 97.463 | 98.830 | 99.763 | 100.000 |

**(b)** Eigenvectors of principle components

|  | F1 | F2 | F3 | F4 | F5 | F6 | F7 |
| --- | --- | --- | --- | --- | --- | --- | --- |
| Var1 | 0.086 | 0.891 | 0.376 | 0.125 | 0.113 | 0.048 | -0.164 |
| Var2 | 0.403 | -0.243 | 0.389 | -0.172 | 0.708 | 0.232 | 0.206 |
| Var3 | 0.379 | -0.293 | 0.620 | 0.073 | -0.539 | -0.044 | -0.297 |
| Var4 | 0.385 | -0.046 | -0.231 | 0.878 | 0.078 | -0.039 | 0.134 |
| Var5 | 0.418 | 0.118 | -0.391 | -0.206 | -0.234 | 0.737 | -0.136 |
| Var6 | 0.427 | 0.025 | -0.334 | -0.230 | 0.230 | -0.521 | -0.573 |
| Var7 | 0.425 | 0.212 | -0.069 | -0.290 | -0.286 | -0.355 | 0.691 |

**(c)** Correlations between variables and factors

|  | F1 | F2 | F3 | F4 | F5 | F6 | F7 |
| --- | --- | --- | --- | --- | --- | --- | --- |
| Var1 | 0.193 | 0.948 | 0.240 | 0.069 | 0.035 | 0.012 | -0.021 |
| Var2 | 0.900 | -0.259 | 0.248 | -0.095 | 0.219 | 0.059 | 0.027 |
| Var3 | 0.845 | -0.312 | 0.396 | 0.040 | -0.167 | -0.011 | -0.038 |
| Var4 | 0.860 | -0.049 | -0.148 | 0.485 | 0.024 | -0.010 | 0.017 |
| Var5 | 0.932 | 0.126 | -0.250 | -0.114 | -0.072 | 0.188 | -0.018 |
| Var6 | 0.954 | 0.027 | -0.213 | -0.127 | 0.071 | -0.133 | -0.074 |
| Var7 | 0.947 | 0.225 | -0.044 | -0.160 | -0.089 | -0.091 | 0.089 |

**(d)** Contribution of the variables as a percent for each factor

|  | F1 | F2 | F3 | F4 | F5 | F6 | F7 |
| --- | --- | --- | --- | --- | --- | --- | --- |
| Var1 | 0.746 | 79.357 | 14.123 | 1.567 | 1.270 | 0.234 | 2.703 |
| Var2 | 16.281 | 5.910 | 15.115 | 2.951 | 50.126 | 5.377 | 4.240 |
| Var3 | 14.364 | 8.581 | 38.485 | 0.528 | 29.021 | 0.196 | 8.825 |
| Var4 | 14.858 | 0.214 | 5.332 | 77.030 | 0.608 | 0.153 | 1.805 |
| Var5 | 17.439 | 1.393 | 15.310 | 4.228 | 5.488 | 54.291 | 1.851 |
| Var6 | 18.273 | 0.064 | 11.162 | 5.302 | 5.281 | 27.115 | 32.803 |
| Var7 | 18.039 | 4.480 | 0.473 | 8.393 | 8.208 | 12.634 | 47.773 |

Note: Var1, *StuPPO1* gene expression level; Var2, *StuPPO2* gene expression level; Var3, *StuPPO3* gene expression level; Var4, *StuPPO4* gene expression level; Var5, relative PPO protein level (RPR); Var6, relative PPO activity (RPPO); Var7, relative browning potential (RBR).
