# Supplementary material for: Reduced polyphenol oxidase gene expression and enzymatic browning in potato (Solanum tuberosum L.) with artificial microRNAs
Source: BMC Plant Biol. 2014 Mar 11;14:62. doi: 10.1186/1471-2229-14-62 (PMC4007649; doi:10.1186/1471-2229-14-62)
Supplement: Additional file 6: Table S2 — Relative expression level of PPO genes in the tuber tissue of potato cultivar Alt-1762 (wild type). [file 1471-2229-14-62-S6.docx]

**Table S2** **Relative expression level of PPO genes in the tuber tissue of potato cultivar Alt-1762 (wild type)**

|  | *StuPPO1* | *StuPPO2* | *StuPPO3* | *StuPPO4* |
| --- | --- | --- | --- | --- |
| tuber tissue | 28.13 ±6.08 | 67.62 ±3.99 | 4.11 ±0.60 | 0.14 ±0.01 |

Note: The relative transcript abundances of PPO genes were calculated using formula: (1 + E_PPO_)^-Ct ppo^/ Sqrt [(1 + E_ef1α_)^-Ct ef1α^ ×(1 + E_cyclophilin_)^-Ct cyclophilin^]. The PCR amplification efficiencies of every individual PCR reaction (in-well) in the microtiter plates were estimated directly from each set of amplification curves using the LinRegPCR algorithm ([Ramakers et al., 2003](#_ENREF_3)). The results were presented as the distribution of different PPO genes in the tuber tissue by percentage. The data were reported as means ± standard deviation of two biological replicates. Three technical replicates were performed on each biological replicate.

**Reference:**

Ramakers, C., Ruijter, J.M., Deprez, R.H. and Moorman, A.F. (2003) Assumption-free analysis of quantitative real-time polymerase chain reaction (PCR) data. *Neuroscience letters***339**, 62-66.
