# Supplementary material for: Reduced polyphenol oxidase gene expression and enzymatic browning in potato (Solanum tuberosum L.) with artificial microRNAs
Source: BMC Plant Biol. 2014 Mar 11;14:62. doi: 10.1186/1471-2229-14-62 (PMC4007649; doi:10.1186/1471-2229-14-62)
Supplement: Additional file 7: Table S3 — Primers and amplicon characteristics of reference genes and PPO genes used in quantification of PPO gene expression in potato tuber tissue measured by qRT-PCR. [file 1471-2229-14-62-S7.docx]

**Table S3 Primers and amplicon characteristics of reference genes and PPO genes used in quantification of PPO gene expression in potato tuber tissue measured by qRT-PCR**

| Primer pair names | Gene name | Gene ID | Primer sequences  (forward/reverse, 5'- 3' ) | Amplicon length (bp) | Sequence identity (%) | Amplification efficiency  (%) ± S.D. | R^2^ |
| --- | --- | --- | --- | --- | --- | --- | --- |
|  | cyclophilin | AF126551 | CTCTTCGCCGATACCACTCC/  TCACACGGTGGAAGGTTGAG | 121 | 99.2 | 0.984 ± 0.006 | 0.9999 ± 0.0001 |
|  | ef1α | AB061263 | ATTGGAAACGGATATGCTCCA/ TCCTTACCTGAACGCCTGTCA | 101 | 100 | 0.873 ± 0.004 | 0.9996 ± 0.0004 |
| PPO35/36 | *StuPPO1* | M95196, M95197 | GACCAGCTTCGTCAAGGACTA/  TTGTCAACGTTCAGGAACACA | 121 | 98-100 | 0.998 ± 0.003 | 0.9927 ± 0.0003 |
| PPO41/42 | *StuPPO2* | U22921 | ATATCGCGACTGTTGATTTCC/  GTCGCACCTTCAATGGAGATA | 133 | 99 | 1.012 ± 0.006 | 0.9966 ± 0.0003 |
| PPO46/47 | *StuPPO3* | U22922 | ATGGCGTAACTTCAAACCAAA/  CCATCTTCGTGAGTGGGAATA | 98 | 98-100 | 0.992 ± 0.006 | 0.9954 ± 0.0004 |
| PPO54/55 | *StuPPO4* | U22923, BG592710 | TCTGGTGCCAAAGAAAGGTAA/  ACAAACAATCCGCAGATTCAA | 96 | 100 | 1.000 ± 0.001 | 0.9997 ± 0.0001 |
