# Supplementary material for: Reduced polyphenol oxidase gene expression and enzymatic browning in potato (Solanum tuberosum L.) with artificial microRNAs
Source: BMC Plant Biol. 2014 Mar 11;14:62. doi: 10.1186/1471-2229-14-62 (PMC4007649; doi:10.1186/1471-2229-14-62)
Supplement: Additional file 9: Table S4 — List of forward primers used for detection of amiRNAs by reverse transcription PCR. [file 1471-2229-14-62-S9.docx]

**Table S4 List of forward primers used for detection of amiRNAs by reverse transcription PCR**

| Name | Sequence (5’ – 3’) | Comment |
| --- | --- | --- |
| oligo 1 | TTGGTGACTGGTGCAATTGAC | forward primer for detection of amiRPPO1 |
| oligo 2 | TTGCTAGCTGGCGGAAGTGAA | forward primer for detection of amiRPPO2 |
| oligo 3 | TTGTTCACTGGGGGGAGTGTA | forward primer for detection of amiRPPO3 |
| oligo 4 | TCATCAACTGGAGTTGAGTTG | forward primer for detection of amiRPPO23 |
| oligo 5 | TAGAACTCGGAGTTCAACCAA | forward primer for detection of amiRPPO234 |
| oligo 6 | AAGAACTCGGAGTTCAACCAA | forward primer for detection of amiRPPO234A |
| oligo 7 | TCAAGCTCATTCGCATTCACA | forward primer for detection of amiRPPO1234 |
| ath5.8S | ACGTCTGCCTGGGTGTCACAA | forward primer for detection of 5.8S rRNA (internal control) |

Note: A Universal RT Primer for synthesis of the first strand cDNA and a Universal PCR primer used as the common reverse primer for PCR were provided with the NCode miRNA First Strand cDNA Module Kit (Catalogue No. MIRC-10, Life Technologies) and not listed in the Table.
