# Supplementary material for: Reduced polyphenol oxidase gene expression and enzymatic browning in potato (Solanum tuberosum L.) with artificial microRNAs
Source: BMC Plant Biol. 2014 Mar 11;14:62. doi: 10.1186/1471-2229-14-62 (PMC4007649; doi:10.1186/1471-2229-14-62)
Supplement: Additional file 10: Table S5 — List of reverse primers used in detection of the cleaved mRNA of amiRNA target in transgenic lines amiRPPO1-12 by 5′-RACE PCR. [file 1471-2229-14-62-S10.docx]

**Table S5 List of reverse primers used in detection of the cleaved mRNA of amiRNA target in transgenic line amiRPPO1-12 by 5’-RACE PCR**

| Name | Sequence (5' → 3') | Comment |
| --- | --- | --- |
| Oligo 8 | ACCARAGTYACCGCAATAGT | Common primers for synthesis of the cDNA from truncated PPO mRNAs |
| Oligo 9 | TCKTCATCTTCCAAWCCAWTRTCC | Common reverse primer for 1^st^- round PCR after RT |
| Oligo 10 | TTRTCAAGCTCATYCGCATTCACA | Common reverse primer for nested PCR round-1 |
| Oligo 11 | ACGAAGCTGGTCTGGTGATAGAGA | Specific reverse primer for detection of *StuPPO1* gene mRNA in nested PCR round-2 |
| Oligo 12 | CATATCTTATGCTACTGAATGTCAAC | Specific reverse primer for detection of *StuPPO2* gene mRNA in nested PCR round-2 |
| Oligo 13 | TGAAGCAGGCCTATTGATGGAAAAT | Specific reverse primer for detection of *StuPPO3* gene mRNA in nested PCR round-2 |
| Oligo 14 | TCATACTTTATTTCATTGAACGTTAGC | Specific reverse primer for detection of *StuPPO4* gene mRNA in nested PCR round-2 |

Note: K stands for nucleotide G or T; R for A or G; W for A or T; Y for C or T. 5' RACE primers (forward) was provided in the FirstChoice RLM-RACE Kit (Life Technologies) and not listed in the Table.
